# Percutaneous versus Surgical Intervention for Severe Aortic Valve Stenosis: A Systematic Review

**DOI:** 10.1155/2021/3973924

**Published:** 2021-05-26

**Authors:** Adelaide Iervolino, Sanjeet Singh Avtaar Singh, Pierluigi Nappi, Francesca Bellomo, Francesco Nappi

**Affiliations:** ^1^Department of Cardiovascular Sciences, Fondazione Policlinico Universitario A. Gemelli IRCSS, Italy; ^2^Department of Cardiothoracic Surgery, Golden Jubilee National Hospital, Glasgow, UK; ^3^Department of Clinical and Experimental Medicine, University of Messina, Italy; ^4^Department of Cardiac Surgery, Centre Cardiologique du Nord de Saint-Denis, Paris, France

## Abstract

Aortic stenosis is a disease that is increasing in prevalence and manifests as decreased cardiac output, which if left untreated can result in heart failure and ultimately death. It is primarily a disease of the elderly who often have multiple comorbidities. The advent of transcatheter aortic valve therapies has changed the way we treat these conditions. However, long-term results of these therapies remain uncertain. Recently, there has been an increasing number of studies examining the role of both surgical aortic valve replacement and transcatheter aortic valve replacement. We therefore performed a systematic review using Ovid MEDLINE, Ovid Embase, and the Cochrane Library. Two investigators searched papers published between January 1, 2007, and to date using the following terms: “aortic valve stenosis,” “aortic valve operation,” and “transcatheter aortic valve therapy.” Both strategies in aortic stenosis treatment highlighted specific indications alongside the pitfalls such as structural valve degeneration and valve thrombosis which have a bearing on clinical outcomes. We propose some recommendations to help clinicians in the decision-making process as technological improvements make both surgical and transcatheter therapies viable options for patients with aortic stenosis. Finally, we assess the role of finite element analysis in patient selection for aortic valve replacement. THVT and AVR-S are both useful tools in the armamentarium against aortic stenosis. The decision between the two treatment strategies should be best guided by a strong robust evidence base, ideally with a long-term follow-up. This is best performed by the heart team with the patient as the center of the discussion.

## 1. Introduction

The evolution of degenerative aortic valve disease into stenosis is a disease that is increasing in numbers and manifests itself as obstruction of the left ventricular outflow tract. The result of the reduced blood flow through the valve results in reduced cardiac output, impaired ability to perform physical exercise, and heart failure that can lead to death from cardiovascular causes. The distribution of aortic stenosis in the population prevalence varies among age groups. Although the prevalence is only about 0.2% in the adult population aged 50 to 59, there is an increased number of cases in octogenarians (9.8%). On the other hand, a survey of the adult population over the age of 75 has shown that the disease occurs with an overall prevalence of 2.8% [1, 2]. In people who have aortic stenosis, there is no increase in mortality. In symptomatic patients, however, the mortality rate is greater than 50% at two years after the onset of clinical signs. To overcome this adverse complication, aortic valve replacement must be performed in a timely manner [3, 4].

More than a decade ago, before the advent of transvalvular therapy reported in the landmark randomized clinical trial (RCT) on the use of transcatheter aortic valve replacement (TAVR) in inoperable/high-risk patients who cannot undergo aortic valve replacement surgery (AVR-S), more than 65,000 patients received surgical aortic valve replacement in the United States [5, 6]. The majority of these had severe aortic stenosis.

Practice guidelines of American and European societies recommend consideration for percutaneous aortic valve replacement or surgical intervention for patients with symptomatic severe aortic stenosis despite the best available and optimal medical treatment [7].

These guidelines, however, do not specify whether percutaneous or open surgery is indicated for aortic valve stenosis because there is genuine equipoise while the long-term benefits of TAVR therapy remain unclear [8]. Several randomized controlled trials have suggested that TAVR is associated with no significant differences in rates of death and repeat hospitalization in high-risk surgical patients compared to standard aortic valve replacement [4, 9]. Advocates for SAVR adduce better long-term survival with significatively lower all-cause mortality [8]. This controversy is primarily based on the lack of data from rigorous RCTs in patients with greater than 5 years of follow-up and with a relatively low STS score [10]. This category of patients could help in determining whether the potential benefits of using the percutaneous procedure outweigh those of the standard surgical operation.

This systematic review is aimed at shedding light on the optimal use of mechanical intervention and clarifying whether large promulgation of the percutaneous procedure is advisable. We discuss the ongoing evidence base for the use of TAVR operation or AVR-S for severe AVS. We also aim to guide healthcare professionals by proposing a useful algorithm for shared decision-making in patients for the treatment of severe aortic valve stenosis.

## 2. Methods

### 2.1. Eligibility Criteria

We systematically reviewed the literature to identify relevant randomized controlled trials (RCTs), propensity-matched observational series, and meta-analyses which were considered initially and followed by unmatched observational series.

Studies were considered eligible for inclusion with the following criteria: adult patients (≥18 years old) with aortic valve surgery or transcatheter aortic valve therapy.

Exclusions consisted of the following: animal or pediatric studies and nonprimary studies (i.e., letters, editorials, and review articles). Papers were also excluded if the authors were unable to obtain a translation or published as an abstract only.

### 2.2. Search Strategy

The search was carried out on December 6, 2020, using the following databases: Ovid MEDLINE (1946 to the present), Ovid Embase (1974 to the present), and the Cochrane Library (Wiley; 1996 to the present).

### 2.3. Data Extraction

**Figure 1:**
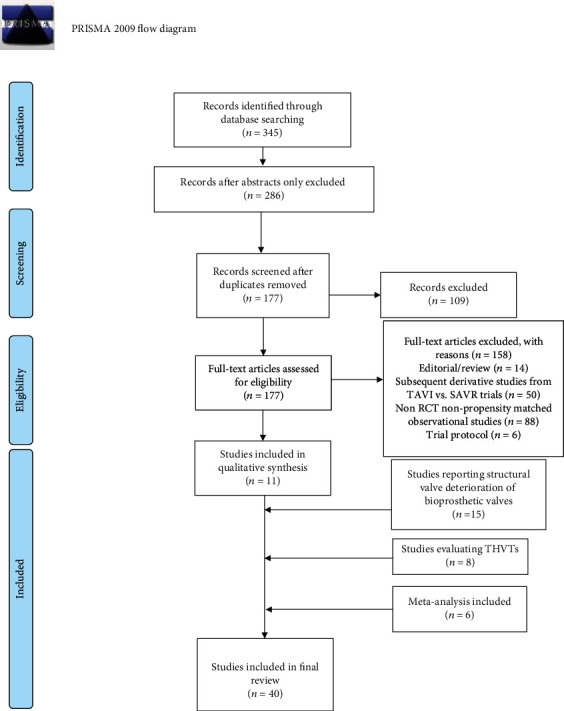
The PRISMA Chart.

Two investigators (A.I. and SSAS) searched papers published between January 1, 2007, and to date using the following terms: “aortic valve stenosis,” “aortic valve operation,” and “transcatheter aortic valve therapy.” These terms were coupled with “standard surgical aortic valve replacement,” “open aortic valve surgery,” “transcatheter aortic valve replacement,” and “transcatheter aortic valve implantation.” For completion, the following terms were added: valve thrombosis, valve dysfunction, valve fibrosis, thromboembolism, stroke, or complications. Randomized controlled trials (RCTs), propensity-matched observational series, and meta-analyses were considered initially and followed by unmatched observational series. Pertinent abstracts were reviewed, and data were extracted independently for all enclosed manuscripts. The correct progression of the study was verified by a third investigator (F.N.) to ensure accuracy. PRISMA Chart is in Figures 1 and PRISMA 2020 Checklist Item is in Table 1.

Extracted data were transposed onto a table with the following subheadings: study completion date, number of patients, follow-up period, primary and secondary endpoints, and study findings.

### 2.4. Risk of Bias Assessment

The Cochrane Risk of Bias assessment tool was used for randomized controlled trials. Assessment of bias of the RCTs showed a low risk of reporting, detection, or attrition bias as the outcomes were well reported and the loss to follow-up was low.

Given the significant heterogeneity in study design across identified studies, formal data synthesis via meta-analysis was not conducted. Hazard ratios (HR) with 95% CIs were calculated where possible for primary and secondary endpoints as shown in Tables 1 and 2.

## 3. Results

### 3.1. Which Patients Were Suited for Mechanical Intervention?

When analyzing the different age groups, it is apparent that majority of the patients who underwent treatment for AVS were over the age of 65, accounting for about 70% of cases [5]. The trend does not differ in the European population [11] resulting in increased costs to healthcare in the aging population among most industrialized countries [12].

The main concern is the lack of suitable medical therapies in preventing or slowing the progression of aortic valve stenosis. Therefore, the primary work required of health providers is in improving disease outcomes in the population at risk, identifying patients at risk for valvular disease, accurately measuring the severity of stenosis, managing any concomitant disease, and ensuring the appropriate timing and the type of replacement of the aortic valve [7, 9, 13].

For surgeons and interventional cardiologists, the range of aortic stenosis assumes significant importance during mechanical intervention. The lesion may appear as a calcified alteration limited to the valve leaflet or extended with penetrating calcifications in the annulus and aortic root. The extent of the lesion can be of different severity evolving to critical valvular obstruction and in the most severe forms to the involvement of the left ventricular outflow tract affected by solid calcium agglomerates. It is important to note that once aortic stenosis becomes severe, it may be without or with clinical symptoms [7, 11, 14]. Patients who are considered for conventional surgery or interventional cardiology may have the complete clinical complements of aortic stenosis severity that has been diagnosed by integrating information on valve anatomy [15, 16], hemodynamics, symptoms, and left ventricular response to pressure overload [7, 11]. Indices commonly used as a synonym for critical aortic valve stenosis incorporate the maximum transvalvular velocity and the mean transaortic pressure gradient. The pattern is variable and the patient can remain asymptomatic as long as the maximum transvalvular velocity is more than four times the normal velocity (i.e., increased to 4.0 m per second). However, severe valve obstruction with a low velocity and pressure gradient involving a small area of the aortic valve may occur in patients with concomitant left ventricular systolic dysfunction. Rarely, patients may have severe low-gradient aortic stenosis even with a normal left ventricular ejection fraction [7, 11].

### 3.2. Transcatheter Aortic Valve Replacement

In April 2009, Webb et al. reported the first large series of transcatheter aortic valve implantation [17]. In the 12 years since then, transcatheter aortic valve therapy (TAVT) has been conceivably the most vigorously studied percutaneous procedure for structural heart valve disease. One of the most significant and resolute controversies emerging in the international guidelines is the ideal choice of the mechanical intervention for severe AVS and, in particular, whether the use of TAVR has resulted in significantly improved long-term outcomes [18–20].

Over the past six years, a considerable number of RCTs demonstrating favorable results of TAVR operation have been published. The majority of these reported survival benefits have occurred with the use of self- and balloon-expandable devices rather than mechanically expanded devices [3, 4, 9, 10, 21–28] (Table 2).

An important element to consider is that up to 2011, even with propensity-matching, most of the studies were tested on retrospective observational data and no suitable randomized comparative studies had been published [29–34].

The better outcomes associated with the use of TAVR were derived as alternatives to conventional surgery in high surgical risk patients which resulted in similar clinical outcomes. In this scenario, the first randomized clinical trials (RCTs) evaluating the percutaneous procedure with self- and balloon-expanded devices showing the superiority of TAVR in high/prohibitive risk patients compared to medical therapy and noninferior results in terms of mortality to SAVR were presented to the American Heart Association in November 2014 [18].

Recent RCTs have steadily revealed that the use of self and balloon TAVT in low-risk patients had a composite rate of death, stroke, or rehospitalization at 1 year that was significantly lower in patients who underwent TAVR compared to their surgical counterparts [10]. Another RCT highlighted the noninferiority of TAVT compared to SAVR at 24 months [26]. Of note, these findings are presently based on a larger number of trials and patients who received either the balloon-expandable (3 trials, *n* = 2366) or the self-expandable (4 trials, *n* = 304) devices.

### 3.3. PARTNER Trial Consortium

The PARTNER consortium (Placement of AoRTic TraNscathetER Valve Trial Edwards SAPIEN Transcatheter Heart Valve) is one of the largest multiple randomized studies on the use of TAVR encompassing the PARTNER 1, 2, and 3 RCTs. The authors involved an average of 50 centers and 4089 patients randomized to receive mostly TAVR or SAVR [3, 4, 6, 9, 10, 13, 21, 35, 36] (Table 2).

The PARTNER 1 RCT [4, 9, 21] enrolled 699 patients with severe AVS and NYHA Class ≥ 2, at high risk for aortic valve replacement surgery (AVR-S), while the PARTNER 2 RCT [35, 36] enrolled 2032 patients with symptomatic severe aortic stenosis at intermediate risk.

The implanted devices follow the advancements in the SAPIEN system, as in the PARTNER 1 RCT investigators used the SAPIEN (Edwards Lifesciences) self-expandable balloon valve system while the devices used in PARTNER 2 were the second-generation balloon-expandable SAPIEN XT heart valve system (Edwards Lifesciences). The latter is different from the first generation of SAPIEN valve as it incorporates thinner cobalt-chromium frames, 29 mm valve size for larger aortic annulus, and a low profile delivery catheter to reduce trauma.

In the PARTNER 1 RCT, transcatheter transfemoral placements and transapical ones were performed based on the size of the peripheral artery chosen as access. A 22 Fr sheath was used for the 23 mm valve and 24 Fr sheath for the 26 mm sized valves, respectively.

Results showed that high-risk patients with aortic stenosis had the same mortality at 1 year, 2 years, and 3 years with the use of either TAVR or AVR-S highlighting a risk of death in 67.8% of recipients in the TAVR cohort compared with 62.4% of the AVR-S one. Also, moderate or severe aortic regurgitation was reported in 14% of patients undergoing operation TAVR and 1% in those receiving AVR-S. A notable consequence of this was the increased risk of mortality at 5 years for patients who had moderate or severe aortic regurgitation after TAVR operation [9].

Thus, the trial demonstrated that TAVR is a valid alternative to AVR-S for patients at high surgical risk. This resulted in a change of the American and European international guidelines with the increasing recruitment of intermediate-risk patients and then low-risk ones and rapid marketing of the SAPIEN XT and SAPIEN 3 products (Table 2).

The PARTNER 2 RCT [35, 36] enrolled patients from 57 centers in the United States and Canada. Patients' risk was assessed by the multidisciplinary heart team (MHT) based on the same risk model generated by the Society of Thoracic Surgeons (STS) to assess the risk of death at 30 days after the surgical procedure. The critical point of the STS risk model was the presence of coexisting diseases that predicted mortality at 30 days. Therefore, the extrapolated STS score was equivalent to the predicted mortality.

Patients scheduled to receive TAVR operation were managed with either transfemoral or transthoracic placement, as in the PARTNER 1 RCT.

The 5-year PARTNER 2 RCT [36] reported no significant difference in the incidence of death from any cause or disabling stroke among patients who underwent TAVR operation compared to those who received AVR-S. Evidence demonstrated that although mortality and disabling stroke were comparable either in the transfemoral access population or in the AVR-S population, the incidence of death or disabling stroke was higher in the transthoracic TAVR group while there was a similar improvement in the state of health in the two populations (Table 2).

The encouraging results for intermediate- and high-risk patients justified the randomized PARTNER 3 trial [10] for patients presenting with severe AVS at low risk for death after surgical procedure. After enrollment, patients were assigned to receive either TAVR operation or AVR-S. The TAVR operation was performed via transfemoral access only, differently from PARTNER 1 and PARTNER 2. Furthermore, the authors used and studied the third-generation balloon-expandable valve (SAPIEN 3 Device). All patients enrolled in PARTER 3 had fewer comorbidities compared to those who participated in previous PARTNER 1 [4, 9, 21] and 2 RCTs [35, 36].

Compared to AVR recipients, the composite of death from any cause, stroke, or rehospitalization at 1 year was lower after the TAVR operation. Importantly, the authors reported a shorter index hospitalization for recipients of TAVR while there were no significant differences between the groups in terms of major vascular complications, new permanent pacemaker insertions, or moderate or severe paravalvular regurgitation. The refined technological advancement has in fact allowed a reduction in the diameter of the introducers and an improved performance of the stent which resulted as safe and effective, although results need to be confirmed by a longer-term follow-up (Table 2).

### 3.4. CoreValve Clinical Investigators Consortium

The US CoreValve Clinical Investigators program was launched as an alternative to the PARTNER trials. The important difference is the transcatheter bioprosthesis consisting of a self-expanding nitinol frame and a porcine trileaflet pericardial valve (CoreValve, Medtronic) [24–26] (Table 2).

The first randomized US CoreValve Clinical Investigators trial (U.S. CoreValve High-Risk Study ClinicalTrials. NCT01240902) [24] enrolled 795 patients undergoing either a TAVR operation by transfemoral and transapical access or an AVR-S. The surgical risk assessment was established on consideration of the Society of Thoracic Surgeons (STS PROM) [37] predicted mortality risk estimate and the impact of frailty status on survival [38]. All patients at increased surgical risk had >15% mortality risk within 30 days after surgery. In the SURTAVI trial, the same interventions were compared in patients with symptomatic, severe aortic stenosis at intermediate surgical risk.

Results from the implantation of the first-generation CoreValve Self-Expanding System show that the 1-year all-cause death rate was inferior in patients after TAVR compared to AVR-S. Echocardiographical parameters demonstrated that TAVR noninferiority and a precise pooled analysis confirmed its efficacy and safety [24].

Given the good results, TAVR operation was enthusiastically accepted by health providers and unquestionably served as an alternative. This has resulted despite increased rates of residual aortic valve regurgitation and a larger number of pacemakers implanted in the population destined for the TAVR procedure. In this direction, a randomized study involving the intermediate surgical risk population was therefore desirable.

The SURTAVI trial [25] was designed as a multinational, randomized, clinical trial. 1660 patients were enrolled to receive either transcatheter aortic valve bioprostheses or AVR-S. Among the former, 84% of patients received the first-generation CoreValve System while 16% the second generation of Evolut R bioprostheses. Transfemoral access was considered the first option. Patients were mostly treated with dual antiplatelet therapy with aspirin (81 to 100 mg dose) and clopidogrel (75 mg). At 24 months, the Kaplan-Meier estimate for the enrolled population presenting an STS score Society for Predicted Risk of Mortality at 4.5 ± 1.6% revealed that the composite of death from any cause or disabling stroke in patients managed with TAVR operation was 12.6% compared to 14.0% in those who received the AVR-S.

The New York Heart Association values were significantly improved in the two populations, and the KCCQ summary score showed improvement in the two populations with the difference that patients managed with TAVR operation had a greater percentage of improvement at 1 month [25] (Table 2).

As the guidelines of the American [19] and European [20] Societies of 2017 recommended the use of TAVR in high-risk patients, in 4 years, it has exceeded in number the conventional surgical replacements. Patients at high surgical risk treated with a self-expanding supra-annular bioprosthesis reported superior results compared to both medical therapy and conventional surgery and no less efficacy and safety in patients at intermediate surgical risk. The use of a self-expanding bioprosthesis in a low-risk population was described in RCT NOTION.

In multinational randomized clinical Evolut Low-Risk Trial Investigators [26] (March 2016 to November 27, 2018), 734 patients with severe aortic valve stenosis were eligible to receive one of three self-expanding, supra-annular bioprostheses (CoreValve, Evolut R, or Evolut PRO; Medtronic), and 734 patients were managed with conventional AV surgery. The entire population has a predicted 3% of STS risk score for death at 30 days with surgery.

At 2 years, the estimated incidence of death or disabling stroke at 24 months was lower for the patients who received TAVR operation than for the AVR-S (aortic valve replacement surgery) recipients (5.3 vs. 6.7%). At 1 year, lower aortic transvalvular gradients after treatment with TAVR were reported in respect to conventional surgery and greater effective orifice areas (2.3 cm^2^ vs. 2.0 cm^2^). The maximum follow-up was 2 years: after 1 year, it was available for 432 recipients of TAVR operation and 352 of AVR-S, respectively; at 2 years, for 72 patients for the TAVR group versus 45 in the AVR-S group. Despite this reduction, TAVR with a self-expanding supra-annular bioprosthesis was demonstrated to be noninferior to conventional AV surgery with respect to the composite endpoint of death or disabling stroke. Doubts arise about the publication of longer follow-up results due to the low number of patients [26] (Table 2).

### 3.5. Transcatheter Heart Valve Thrombosis

**Figure 2:**
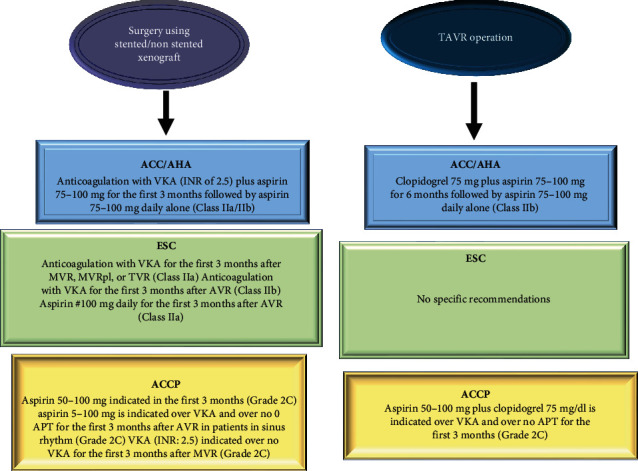
ACC/AHA, ACCP, and ESC recommendations for antithrombotic therapy in patients receiving TAVR operation or AVR-S with bioprostheses.

Evidence based on multiple studies has shown that patients undergoing TAVR have an uncertain incidence of bioprosthetic valve thrombosis (BPV-TH) and thromboembolic complications. In this regard, the results of the PARTNER and CoreValve System randomized trials did not report cases of BPV-TH [9, 13, 39]. The EU PARTNER Registry [40] also produced very poor data on thromboembolic complications in patients undergoing transcatheter procedures describing only one case of BPV-TH out of 130 patients recruited for mechanical intervention. In a multicenter study that enrolled a large number of patients (4266) managed with TAVR, only 27 cases of BPV-TH thrombosis (0.61%) were reported, all occurring within a median of 181 days (interquartile range: 45 to 313 days) after device implantation [40]. This data provides preliminary data that the risk of thromboembolic complications and thrombosis of the bioprosthesis after the percutaneous transcatheter mechanical intervention was greater in the first 3 months after the procedure; the risk curves extend for a marked reduction in the following months almost matching the curves of the general population [41]. There is a clear relationship between the physiological processes related to neointimal proliferation and the time in which this is defined in its entirety. Indeed, histopathological studies performed on the CoreValve device suggest that this process was completed approximately 3 months after the CoreValve System was implanted [42–46]. It seems evident that both the use of mechanical or stented conventional xenograft after conventional surgical approach and that of bioprosthetic valves in transcatheter procedures have noted that the incidence of thrombus formation, with or without prosthetic valve dysfunction, was likely influenced by either intensity with which the neointimal proliferation process proceeds or from the time of screening [44].  Figure 2 reports current guidelines for antithrombotic therapy after surgical valve replacement with bioprosthesis or TAVR.

A contribution to the clarification of the TH phenomenon was given by the systematic use of computed tomography for images by Makkar et al. [47]. The data emerging from the analysis of 55 patients enrolled in the Portico Studio IDE (Portico Resheathable Transcatheter Aortic Valve System US IDE Trial) showed a median of 32 days after valve implantation, noting reduced movement for 40% of recipients. In 132 patients enrolled in the Savory study (subclinical aortic valve thrombosis assessed with 4D CT), who received either a TAVR or AVR-S operation using a conventional stented xenograft, or included in RESOLVE (surgical catheter and aortic evaluation of thrombosis of the bioprosthetic valve and its treatment with anticoagulation), computed tomography performed within 3 months showed reduced leaflet motion in 13% of recipients, of which 14% had received a transcatheter bioprostheses while 7% had received AVR-S with the use of a conventional bioprosthesis [47, 48].

The concern of reduced leaflet motion appears to play a central role regardless of using both transcatheter procedure and conventional surgery. In fact, a lower prevalence of hypoattenuated leaflet motion was noted in anticoagulated patients. The two populations studied with ongoing early anticoagulant treatment demonstrated a clear resolution of the reduced motion of the valve leaflets in subsequent remote clinical checks. This evidence strongly supports the concept that the hypoattenuated leaflet motion was the prodromal phenomenon of thrombotic complications [47, 48]. It raises concerns underlined by the analysis of pooled registries and observational series, that the risk of stroke or transient ischemic attack occurred with a higher incidence in patients with reduced leaflet mobility than in those without [47–49].

A study performed on 156 consecutive patients receiving TAVR operation using the SAPIEN 3 (Edwards Lifesciences, Irvine, California) supported the results provided by Makkar et al. The authors demonstrated, using multidetector computed tomography performed at a median of 5 days after mechanical intervention, that 10.3% of TAVR recipients (*n* = 10) showed leaflet thickening with hypoattenuation. Although the relevant finding was the absence of symptoms for these patients, they nevertheless exhibited a higher mean transvalvular gradient and anticoagulant drug therapy led to complete resolution of leaflet thickening [50]. A relationship between increased transvalvular gradient and uncontrolled neointimal proliferation with thickening of the leaflets involved patients who received dual antiplatelet therapy (DAPT) less frequently than those on a single antiplatelet drug (37.5% and 50%, respectively). The difference that occurred in the two populations was not conclusive because statistical significance was not reached [50].

Three recent studies have significant relevance in this matter. Hansson et al. [43] focused on the incidence and predictors of BPV-TH in recipients of TAVR operation with the use of balloon-expandable valves (Edwards SAPIEN XT or SAPIEN 3 valves). Patients were monitored by means of transthoracic or transoesophageal echocardiography and multidetector computed tomography to screen for BPV thrombosis at 1-3 months. 7% of patients had evidence of thrombosis at the scheduled check-up with multidetector computed tomography. For these, in 18% of cases, the BPV-TH occurrence manifested with clinical complications. Cox's multivariate regression analysis showed that the 2 independent predictors of BPV-TH after the use of the TAVR procedure were the absence of warfarin as a treatment and larger BPV sizes in the device (valve size 29 mm).

The single-center study of Nührenberg et al. [51] identified hypoattenuated leaflet thickening as a potential precursor of thrombosis. The authors evaluated the association between platelet reactivity and HLAT following transcatheter aortic valve replacement. All patients, including those with oral anticoagulation treatment, had dual antiplatelet therapy with aspirin and clopidogrel for at least 24 hours before the procedure. In patients who had preexisting indications for oral anticoagulation treatment, aspirin was discontinued but the therapy was continued after TAVR for all the rest. Recipients were checked for platelet function, and 4D computed tomography was performed 5 days after valve implantation to determine the association between baseline platelet reactivity and hypoattenuated leaflet thickening. The most important finding of this study showed an 18% incidence of hypoattenuated leaflet thickening with lower complication rates for patients treated with oral anticoagulation. The authors concluded that patients with dual antiplatelet therapy (aspirin and clopidogrel) did not change the onset of early hypoattenuated leaflet thickening [51].

In the GALILEO 4D RCT [52], 231 patients were enrolled and randomly assigned to receive long-term anticoagulation for antithrombotic strategy either with the use of rivaroxaban (10 mg) coupled with aspirin (75 to 100 mg) once daily or with administration of dual antiplatelet-based strategy based on the use of clopidogrel (75 mg) plus aspirin (75 to 100 mg) once daily. Patients had to have undergone successful TAVR and not have any indication for long-term anticoagulation. They were evaluated by four-dimensional CT with scheduled checks at 90 ± 15 days after randomization. The primary endpoint of the study was the percentage of patients who presented with at least one prosthetic valve leaflet with grade 3 or higher motion reduction. This process had to involve more than 50% of the leaflet. Results showed that 2.1% (2 of 97) of patients who received rivaroxaban had at least one prosthetic valve leaflet with grade 3 or higher motion reduction compared to 10.9% (11 of 101) in the dual antiplatelet protocol (difference, −8.8 percentage points; 95% confidence interval (CI), −16.5 to −1.9; *p* = 0.01). Thickening of at least one leaflet occurred in 12.4% (12 of 97) of patients in the rivaroxaban arm compared to 32.4% (33 of 102) in the dual antiplatelet therapy arm (difference, −20.0 percentage points; 95% CI, −30.9 to −8.5). Concerns about the risk of death or thromboembolic events and the risk of life-threatening, disabling, or greater bleeding occurred with a higher incidence in patients included in the rivaroxaban arm (hazard ratios of 1.35 and 1.50, respectively) [52].

Table 3 reports studies evaluating THVT [43, 47–53].

### 3.6. Biomechanics and TAVR Thrombosis

One of the concerns regarding TAVR includes the degree of native valve calcification and its location, stent deformation, and the size of the patient's annulus. Moreover, physiological blood dynamics is a domain that has not yet been fully explored [54].

**Figure 3:**
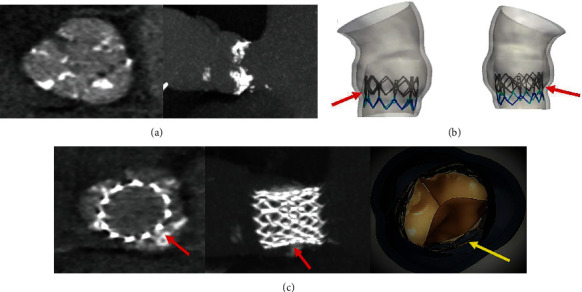
Biomodeling of SAPIEN XT starting from the CT scan. (a) CT scan showed calcifications of aortic valve and root; (b) modeling of the balloon-expandable system showing an incomplete deployment (red arrow); (c) thrombotic formation (red arrow) in correspondence with the distortion of the stent and reduced mobility of the leaflet of the bioprosthesis (yellow arrow).

The calcified blocks in the aortic annulus may have a different consistency thus raising questions on the indication of the use of prostheses with stents that can lead to geometric modifications of the aortic annulus after the deployment of the self- and balloon-expandable systems [55, 56]. We noted that either balloon- or self-expandable catheter-based aortic valves may be ineffective on solid and bulky native aortic valve calcifications. We studied the different degrees of deformation of the SAPIEN XT which showed high values of maximal principal stress in the aortic regions close to solid calcific blocks with consequent deformation of the stent thus assuming an elliptical shape [55] (Figures 3(a) and 3(b)). The concerns related to the geometric modification in the most accentuated form can lead to leaflet malcoaptation characterized by paravalvular leakage or hypoattenuated leaflet thickening (Figure 3(c)). The second type of elliptical deformation suggests a potential predisposition to subclinical thrombosis due to the presence of residual calcifications that favor hypomobility [55]. The SAPIEN 3 Device is highlighted in Figure 3(c) (Figures 3(a)–3(c)).

The system that hinges on self-expansion is not exempt from mechanical distortion phenomena. In fact, in self-expanding devices, we highlighted the crucial role of positioning in determining valve anchorage. Nonuniform expansion related to extensive calcifications is responsible for prosthetic device deformation that leads to an eccentricity > 10%, resulting in incomplete expansion of the metallic frame at almost all levels [56].

It is important to note that the current progress of technology and design associated with advanced clinical studies probably continues to collide with the initial concept that transcatheter heart valve replacements were conceived for use in the pulmonary artery [57], which has a higher degree of extensibility and distortion compared to the aortic root [58]. This essential feature does not apply to the Valsalva sinus of the aortic root, where the predisposition to receive material such as stents is different in both nitinol constituents of the self-expanding valves [55] or cobalt-chromium that integrates the balloon-expandable devices [56]. Thus, thrombus formation may be related to the frame of the prosthesis or stasis in the sinus of Valsalva. Currently, the pathophysiological process accountable for transcatheter heart valve thrombosis remains unseizable. The process is likely mediated by the formation of platelets or thrombin-related clots.

At present, the mechanisms that support thrombosis in TAVR are unknown as well as it is not clear whether the presence of not completely crushed calcifications can favor the formation of clots. The use of finite element analysis is a crucial method for obtaining valuable data on complicated real-world systems; otherwise, it would be impossible to determine immediately. Therefore, its application can lead to the development of predictive models useful for the understanding of the thrombotic process and TAVR functionality in a complex structure such as the aortic root. Mechanical stress can induce remodeling phenomena in the aortic root that can lead to changes in the morphology of the wall [59–75].

### 3.7. Thrombosis in Surgical Prosthesis

The use of conventional stented/nonstented xenograft requires anticoagulation with AVK which is generally recommended for 3 months after the operation. The INR value must fluctuate between 2.0 and 3.0 and is independent of the location of the biological prosthesis (aortic, mitral, or on the right side of the heart) [11, 18, 76]. Once the surgery has been performed with the use of a conventional biological prosthesis, the administration of anticoagulants is aimed at reducing the risk of thromboembolic complications during the endothelialization process.

The large study by Brennan et al. [77] from the Society of Thoracic Surgeons (STS) Adult Cardiac Surgery Database evaluated 25,656 patients aged 65 years who were managed with surgical aortic valve replacement using the conventional stented/nonstented xenografts. The authors reported a marked reduction in the adjusted risk of death (relative risk (RR): 0.80; 95% confidence interval (CI): 0.66 to 0.96) and in the rate of thromboembolic events (RR: 0.52; 95% CI: 0.35 to 0.76) for patients receiving warfarin plus aspirin therapy versus those using aspirin alone. Considering the patient populations studied, it is important to emphasize that there were no significant differences in mortality rates, thromboembolic complications, or bleeding in the aspirin alone population versus the population receiving warfarin alone. These results suggested a greater efficacy obtained by the combining effect on the reduction of platelet activity and the coagulation cascade. Another study [78] showed similar results with the use of aortic bioprostheses. At a 3-month follow-up, the warfarin-treated group had a significantly lower risk of TE, stroke, and cardiovascular death than those receiving aspirin. The benefit of the use of warfarin was even more evident between 3 and 6 months with an increase in thromboembolic complications for patients with discontinuation of VKA-based therapy. The paucity of data based on prospective, randomized controlled studies does not allow us to draw firm conclusions about the optimal duration and intensity of VKA therapy after surgical replacement with the use of conventional stented/nonstented xenograft. The available evidence is largely provided from registry studies which, although numerically large, have their intrinsic constraints.

The model of bioprosthesis used is to be expected to potentially affect the choice of treatment with the optimal antithrombotic therapy. The results reported in a meta-analysis based on the selection of randomized and nonrandomized patients at follow-up showed that the population who received conventional nonstented xenografts was associated with a lower risk of prosthesis-patient mismatch, increased effective orifice areas, and lower transvalvular gradients, compared to stented bioprostheses [79]. It is indisputable that hemodynamics is improved in conventional nonstented xenograft thereby reducing the risk of unfavorable thrombotic complications, although more robust studies are lacking. Patients in whom nonstented bioprosthetic models have been used can avoid more intensive antithrombotic therapy. However, evidence based on randomized trials designed with adequate power is required to confirm these data.

### 3.8. Structural Valve Deterioration in Mechanical Intervention

The term SVD is used to indicate a fundamental and acquired abnormality of the valve bioprosthesis caused by a deterioration of the leaflets and the intrinsic structural support of the device resulting in thickening, calcification, tearing, or rupture of the materials that constitute the valve prosthesis. In this context of anatomopathological anomaly, any associated valvular hemodynamic dysfunction, such as the development of stenosis or regurgitation, may manifest. The precise mechanisms underlying an SVD are not yet fully understood. However, they likely include multiple components, mechanical and fluid dynamics, which lead to the rupture or thickening of the tissues over time. An important role is played by mechanical stress in combination with flow anomalies combined with shear stresses on the surface of the valve leaflets that influence the progression towards SVD where there is collagen breakage of fibers and calcification of tissues. There are other clinical conditions due to valve abnormalities that do not have the pathological features of structural deterioration of the valve tissue that deserve consideration and therefore cannot be categorized as part of SVD definition. These inherent alterations to the bioprosthesis include patient-prosthesis mismatch, device malposition, paravalvular regurgitation, and abnormal frame expansion. It is important to underline that these anomalies attributable to the implanted bioprosthesis can be associated with early SVD or be regarded as a cause for its development. Particular attention should be given to the patient-prosthesis mismatch that can hardly be distinguished from a structural degeneration of the valve. Although it is not considered an SVD because the leaflet morphology is normal, the valve area is relatively small and with a high gradient. The main factor that allows differentiating the prosthetic mismatch and the SVD is the time during which the anomaly is established. In the case of patient-prosthesis mismatch, the hemodynamic anomalies of the valve appear at the moment of implantation of the prosthesis, and the deterioration of the patient's hemodynamics manifests itself with an increase in the gradients and decrease in the valve area, which ultimately lead to increased criticality highlighted by repeated echocardiographic checks. In the case of SVD, the associated stenosis is progressively acquired and manifests itself in a much more nuanced way during the follow-up. Prosthetic valve thrombosis and infective endocarditis are not included in the definition of SVD although can subsequently lead to SVD despite being treated successfully.

The literature that addresses the problem of structural valve degeneration (SVD) of bioprostheses is vast. It mainly consists of a large number of observational studies that report the duration of single models of biological valves. Since there is no unanimous definition of SVD, this has resulted in a major obstacle when comparing the durability of diverse biological prostheses. Although freedom from reoperation was considered the primary endpoint in many reports, SVD was identified with the need for repeat surgery; however, this is a poor expedient for SVD because repeat surgery with the use of the conventional procedure or with percutaneous intervention may be necessary for reasons unlike the onset of SVD.

There are two other points to consider. The first concerns the problem that reoperation may not be performed because the SVD went unnoticed at the echocardiographic examination. The second point concerns the clinical conditions of some patients with older generation bioprosthetic valves who have multiple comorbidities and for whom a new intervention is deemed too high risk. These indicate that repeat surgery rates could underestimate the rate of structural valve degeneration of bioprostheses. The debate on SVD has had significant impetus since this complication could influence the choice towards a TAVR procedure. Indeed, since a less invasive transcatheter approach is available for patients presenting with comorbidities and at high risk for the conventional surgical strategy, the relatively short time to reoperation observed could be explained. For many cardiologists following these criteria, it is evident that SVD is not a reliable criterion for establishing true biological valve durability. They found that the reported actuarial freedom from reoperation is intrinsically inferior to freedom from SVD.

The following are the 2009 guidelines of the American Society of Echocardiography [80] for the evaluation of the bioprosthesis aortic valves establishing the presence of possible stenosis: the peak velocity of the prosthetic aortic jet redefined from 3 to 4 m/s, an average gradient from 20 to 35 mmHg, and an effective orifice area from 0.8 to 1.2 cm^2^. Stenosis was defined as significant in the presence of a prosthesis with peak aortic jet velocity > 4 m/s, mean gradient > 35 mmHg, and effective orifice area < 0.8 cm^2^ [80]. Considering the recommendations of the VARC-2, the useful elements to define SVD as valve-related dysfunction were the mean aortic gradient ≥ 20 mmHg, the effective orifice area ≤ 0.9-1.1 cm^2^, a dimensionless valve index < 0.35 m/s, and moderate or severe prosthetic regurgitation. Alternatively, there is recommendation to perform a repeat surgery by means of mechanical intervention with either TAVR operation or AVR-S [81]. For the European Association for Cardiovascular Imaging, the suggestion was to incorporate an increase in mean gradient at the subsequent echocardiographic follow-up with obstruction at the level of prostheses as the main element. Therefore, indicators of SVD were possible obstruction with an increase in gradient mean of 10–19 mmHg during follow-up and significant obstruction when an increase of ≥20 mmHg occurred [82]. More recently, the increase in mechanical intervention procedures using a TAVR operation convinced the European Association of Percutaneous Cardiovascular Interventions to modify the recommendations thus suggesting a distinction between hemodynamic and morphological SVD [83].

**Figure 4:**
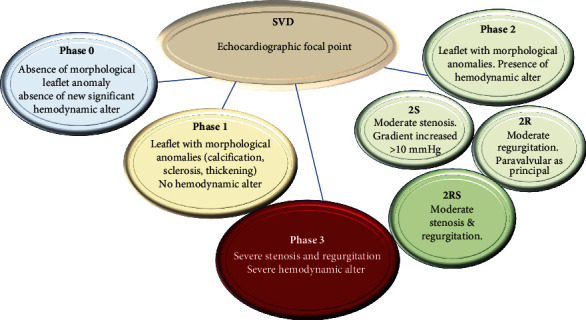
Echocardiographic focal point of the SVD of the stent/stentless xenograft. Infective endocarditis, valve thrombosis, and isolated patient-prosthesis mismatch without functional valve deterioration, isolated paravalvular regurgitation, and frame distortion without abnormal leaflet function are excluded at this focal point of SVD. However, these conditions may explain phase 1 SVD because these bioprostheses could be inclined to early SVD.

The echocardiography has enabled us to evaluate with accuracy the evolution of morphological abnormalities related to the SVD with identifications of different internships (Figure 4). In phase 1, early morphological changes of the leaflet may be noted in the absence of hemodynamic posthumous. Echocardiographic examination highlights the morphological abnormalities of the bioprosthesis as leaflet thickening, leaflet fluttering, and asymmetrical leaflet opening or closure. The morphological alterations typical of stage 1 are also referable to prostheses where the degenerative process is controlled using antithrombotic drugs that reduce the thickening of the leaflet. This is the case, where an already established degenerative process leads the leaflets to a recurrent thickening resulting in an accelerated process of SVD. These bioprostheses require careful surveillance and more frequent follow-up. Morphological abnormalities of valve leaflets in phase 2 of SVD are coupled with hemodynamic dysfunction. The bioprostheses in this phase can manifest as stenosis or regurgitation which in different ways accelerate the rate of deterioration by interfering with the clinical implications and modalities of failure. Experts in the field have ruled out thrombosis as a factor favoring phase 2 since it is the source of the stenosis or paravalvular leakage that leads to the regurgitation of the bioprosthesis. Moderate stenosis or moderate regurgitation allows for the classification of bioprostheses exhibiting typical stage 2 changes into two subcategories, phase 2S and phase 2R, respectively. Bioprostheses with stage 2S degenerative evolution have an increase in the mean transvalvular gradient of ≥10 mmHg with an associated decrease in the valvular area, although a lone leaflet thickening does not occur [84].

An important consideration concerning many of those valve bioprostheses that have baseline postprocedural gradients between 15 and 19 mmHg accrued from a mismatch between prosthesis and patient is that the slight increase in gradients noted upon examination is often related to an increase in blood flow which, however, should not be interpreted as the evolutionary phase of an SVD. A confusion that emerges from an imprecise evaluation leads to an improper finding of abnormal leaflet morphology and deterioration of valvular hemodynamics for increased gradients and decreased valve area, which is decisive for the interpretation of structural valve deterioration [84].

It is not unusual for a bioprosthesis to have a combination of moderate stenosis and moderate regurgitation that take the form of mixed 2R and 2S forms. These should be rated differently from isolated stenosis or isolated regurgitation [85]. Some patients who experience stage 2 SVD have symptoms that meet recommendations for repeat surgery [84].

The most severe extreme spectrum of SVD is represented by severe stenosis/severe regurgitation. These bioprostheses occupy phase 3. The multiplicity of echocardiographic data processed did not appropriately distinguish phase 3 SVD in severe stenosis or severe regurgitation. This is because regardless of the different hemodynamic degradation of SVD in stage 3 (stenosis or regurgitation), patients with bioprostheses at this stage are symptomatic and generally require mechanical intervention. In the future, progressive stratification of growing stage 3 lesions would be advisable for more accurate patient staging [84].

### 3.9. Structural Valve Deterioration of TAVR

Five-year results of surviving patients (median follow-up 3.14 years, IQR 0.68-4.92) enrolled in PARTNER 1 RCT (656/697; 94%) showed that valve hemodynamics was similar in both TAVR or AVR-S interventions and no structural deterioration of the valve was noted requiring repeated surgical valve replacement in both populations. A more detailed longitudinal statistical analysis on the surviving patient population disagreed with these results, pointing out that a percentage of patients underwent repeat surgery. In detail, repeat surgery was required for 5.7% of the recipients of a TAVR operation (*n* = 20) compared to 0.3% of those who received AVR-S (*n* = 1). In the TAVR population, SVD occurred for 25% of cases (*n* = 5) while in the majority of patients it was determined by the development of a paravalvular leak. A previous study showed freedom from SVD and reoperation at 4 years with a substantial increase of moderate prosthetic valve failure (3.4%) that occurred in living patients (9.7%) in the last fifth without requiring either valve-in-valve procedure or conventional repeat aortic valve surgery [9].

The data from PARTNER 2 RCTs are more accurate regarding reoperation and the occurrence of SVD. Although failure related to aortic valve reoperation was rare for both patients who received a TAVR operation and recipients of an AVR-S, however, it was more frequent after percutaneous treatment (*n* = 21) than with conventional surgery (3.2% vs. 0.8%; hazard ratio, 3.28; 95% CI, 1.32 to 8.13). Reoperations after TAVR occurred for progressive stenosis (10 of 21 patients) or aortic regurgitation (11 of 21 patients). All patients (18 of 21) were treated with a new percutaneous procedure with repeat TAVR or balloon valvuloplasty [22].

In patients who received the third generation of the Medtronic CoreValve System, midterm prosthesis failure occurred with a rate of 1.4% at 5 years. In the SURTAVI RCT with a 2-year follow-up, there were no cases of SVD in patients at intermediate surgical risk who received the CoreValve bioprosthesis (84%) or the Evolut R (16%) [25].

In the NOTION RCT at the 6-year follow-up, the authors report that SVD rates were significantly higher after AVR-S than TAVR operation (24.0% vs. 4.8%; *p* < 0.001). Postprocedural echocardiographic controls showed a mean gradient of >20 mmHg in 22% of patients treated with AVR-S compared to 2.9% for those receiving TAVR (*p* < 0.0001). This trend was also confirmed 3 months postprocedure when considering a modified definition of SVD, fixed by a mean gradient increase > 10 mmHg (AVR-S 12.4% vs. TAVR 1.4%; *p* < 0.001) [86].

### 3.10. Structural Valve Deterioration of Bioprosthetic Surgical Valves

The comparison on the durability of the different models of bioprosthetic valves implanted over the last 50 years suffers from the absence of randomized multicenter studies that have been completed by enrolling large numbers of patients. In fact, the main obstacle is the absence of comparison between the different implanted bioprostheses and the fact that the longer duration follow-up studies mainly consist of observational cohort studies. A decisive example emerges from the data coming from the use of the first-generation valve conceived with pericardial tissue, the Ionescu-Shiley, which demonstrated how the different models of valves implanted in the first 15 years of their use in cardiac surgery are not the same with regard to longevity [87]. Ionescu-Shiley demonstrated efficacy and safety with excellent hemodynamics in the first 5 years after implantation. SVD problems subsequently emerged related to the suture fixation of the leaflets which led to the rupture of the cusp and aortic regurgitation. Only 38% of bioprostheses of this type used were intervention-free at 13 years [87].

A contemporary meta-analysis including patients receiving either aortic stented or nonstented xenograft (all types of porcine and pericardial) showed that SVD usually initiated 8 years after surgery, with a greatly increased rate of SVD after 10 years [88–90]. It has also been found that these results are comparable to those of patients where cryopreserved aortic homografts were used as biological substitutes [91–93]. Likewise, the use of St Jude Toronto SPV stentless aortic bioprosthesis (St Jude Medical) demonstrated promising excellent results for hemodynamics and durability up to 5 years after implantation; however, a high rate of SVD occurred within 8 years due to increased mechanical stress on the leaflets associated to the development of late dilatation of the sinotubular junction [94].

Schaefer et al. [95] compared patients receiving CEP stented with those who had AVR-S with Sorin Freedom Solo stentless aortic valve (SFS) (LivaNova PLC, London, UK) at follow-up duration exceeding a mean of 6 years. The authors reported a significant reduction in SVD (0% vs. 5.2%; *p* = 0.04) in the CEP population compared to SFS. The hemodynamic superiority for recipients of the SFS was noted with significantly lower postoperative peak and mean pressure gradients compared to those who had the CEP (*p* < 0.001).

A further type of bioprosthesis widely used is second-generation porcine Hancock II bioprostheses (Medtronic). Nishida et al. [96] reported favorable midterm failure rates in patients using either porcine or bovine bioprostheses that were relatively poor for patients >65 years old (<1% before 5 years and 10% at 10 years). David et al. showed that long-term outcomes including actuarial survival rates without SVD at 10, 15, and 20 years were 95%, 75%, and 49%, respectively, [97, 98]. Glaser et al. reported no significant difference for freedom from reoperation due to SVD at 8 years in recipients Carpentier-Edwards Perimount (Edwards Lifesciences Inc., Irvine, California, USA) compared to those who received Hancock II bioprostheses (98.0% and 97%, respectively; *p* = 0.745) [99].

Landmark studies reported that Carpentier-Edwards Perimount stented pericardial valve (CEP) in the aortic position has been largely used by the surgical community since the 80s [100, 101]. A vast proportion of patients in North America and Europe currently underwent AVR-S with this type of stented xenograft. Bourguignon et al. [100] evaluated 2559 patients who underwent AVR-S with the use of CEP of which 13.9% of recipients were less than 60 years. The authors reported a 48.5% rate of patients showing actuarial freedom from SVD at 20 years with a follow-up duration greater than 10 years. Importantly, these patients had an expected valve duration with a median SVD-free survival time of 19 years.

Johnston et al. [101] evaluated 12,569 with a mean follow-up duration exceeding 5.8 years and receiving AVR-S with the use of CEP in the aortic position. The bioprostheses were explanted from 354 recipients with a rate of 44% in which SVD occurred while for 41% the cause of repeat surgery was related to endocarditis. Of note, actuarial freedom from SVD was 45% (95% CI, 39 to 52) for patients younger than 60 years and 8.1% (95% CI, 6.7 to 9.7) for patients 60 to 80 years old at 20 years.

For the latest generation of prostheses, solid long-term results have not yet been achieved, so the newer generation models of pericardial valves are supported by only midterm results. For patients who had Mitroflow bioprosthesis (models 12A/LX; LivaNova), the mean time to SVD was only 3.8 ± 1.4 years [102]. Substantial long-term data are also not available for the St Jude Trifecta aortic bioprosthesis. Indeed, this valve showed actuarial freedom for SVD and freedom from reoperation of 95% and 96%, respectively, at 6 years in controlled patients [103]. However, unfavorable results have been described in patients in whom early Trifecta failure occurred [104].

Efficacy and safety in duration for bioprosthetic valves without surgical sutures LivaNova Perceval and Edwards Intuity are supported, according to updated data available in the literature, only on short-term outcomes [105, 106], although rare reports have been received that described early failure and showed leaflet flicker in recipients of a Perceval prosthesis [107]. SVD of different models of bioprosthesis are reported in Table 4.

## 4. Discussion

Mechanical intervention for the correction of severe aortic stenosis is aimed at relieving symptoms, improving exercise capacity and quality of life, and extending life expectancy. For patients who have received the intervention, indirect physiological benefits related to the procedure can be summarized in the improvement of the left ventricular function and the regression of the left ventricular hypertrophy. Patients who require earlier intervention may be assessed by results of exercise testing, biomarkers, rapid progression, or the presence of very severe stenosis.

The most important consideration that needs to be worked out when planning AVR-S or TAVR operation is the overall life expectancy of the patient referred for the procedure. The intervention must be considered regardless of the patient's age when the overall life expectancy is greater than 1 year, or there is a likelihood of 25% survival for the recipient in the presence of an improvement in clinical symptoms for 2 years from the procedure. A multifactorial approach is required to determine the degree of risk of the procedure to be undertaken and the choice of intervention to be used for patients who require mechanical intervention. Additional evaluations to be explored include the coexistence of coronary artery disease, the presence of other valve disorders, and noncardiac comorbid conditions. Among other things, the overall life expectancy, the fragility of the patient, and the results of invasive and noninvasive anatomical tests take on a decisive significance [7, 20].

Today, all of these assessments are best handled by the multidisciplinary group of the heart team which is made up of valve cardiologists, imaging specialists, interventional cardiologists, cardiac surgeons, and anesthesiologists. Sometimes, doctors who have gained experience in the treatment and evaluation of the pathologies of the elderly are added to these groups. The heart team can collectively perform a risk-benefit analysis addressed to the individual case and ultimately address the choice for the best option for aortic valve replacement. In addition to the patient's preference for choosing the type of mechanical intervention, their families are also included in this overall assessment necessary for decision-making [7].

**Figure 5:**
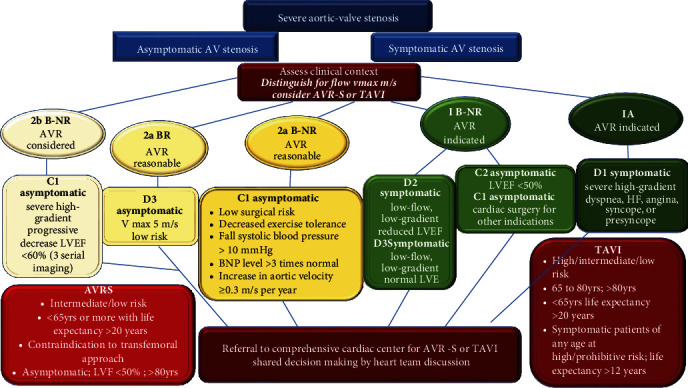
Recommendations from the 2020 guidelines of the American College of Cardiology and the American Heart Association for the treatment of patients with valvular heart disease. Clinical factors and imaging findings are shown in green and yellow boxes as well as AVR recommendations according to class (strength) of recommendation and level (quality) of evidence. Treatment recommendations are shown in red boxes. 1A, 1B-NR, 2aB-NR, 2a B-R, and 2b NR are the class of recommendation (COR) that indicates the strength of recommendation, including the estimated magnitude and assurance of advantage in relation to risk. The level of evidence (LOE) rates the quality of scientific evidence supporting the intervention on the basis of the type, quantity, and consistency of data from clinical trials and other sources. Ref. [7, 19, 20, 118–150]. Abbreviations: AV = aortic valve stenosis; AVR-S = surgical aortic valve replacement; HF = heart failure; LVEF = left ventricular function; TAVR = transcatheter aortic valve replacement.

Conventional surgical aortic valve replacement is considered the standard approach in the 2020 AHA/ACC international guidelines [7] for patients presenting with or without clinical symptoms with severe AVS and any indication for AVR-S, aged <65 years, or having a life expectancy > 20 years (COR 1/LOE A) [90, 108, 109]. Highly relevant meta-analysis showed that AVR-S is recommended in preference to TAVR operation for patients without symptoms presenting with very severe AVS or severe AS with rapid progression. Other important indicators for AVR-S in this category of patients are elevated BNP and abnormal exercise test (COR 1/LOE B-NR). The authors also focused attention on valvular or vascular anatomy as a limiting factor for the transfemoral approach which is not suitable in favor of AVR-S that is recommended with the use of the bioprosthetic valve (COR 1/LOE A) [90, 108–110] (Figure 5).

It should be noted that for the standard surgical approach, the overall mortality at 30 days is less than 3% for isolate aortic valve replacement and approximately [37] 4.5% to replace the aortic valve with coronary artery bypass grafting [38]. However, the most important result to be achieved, which fully justifies the success of AVR-S, is that after recovery the overall survival rate is similar to the matched population without severe AS.

Mechanical intervention performed with the TAVR procedure was initially recommended in patients presenting with symptoms of severe aortic stenosis for whom surgery was high risk. For patients who were deemed to receive an AVR-S, the STS high-risk score was defined as an expected risk of death or major complications with surgery of more than 50% at 1 year. They had a clinical picture that involved three other main organ systems, with a high unlikelihood of improving their clinical condition after surgery with a traditional open approach because these patients could have a severe form of calcification of the aorta, structurally fragile with a high risk of rupture (“porcelain aorta”). It is important to note that TAVR operation is recommended as an alternative to AVR-S if predicted postpercutaneous survival is >12 months with an acceptable quality of life (COR 1/LOE A) [6, 7, 9, 13, 24, 111] (Figure 5).

The use of the TAVR operation is recommended by current guidelines and position papers of professional societies for symptomatic patients with severe AHV aged 65 to 80 years, for those over 80 years old, or younger recipients with a life expectancy < 10 years. For these last two categories, TAVR operation is preferable to conventional surgery [7]. These patients did not have any anatomic contraindication to transfemoral access and in consideration of the rigorous balance between expected patient longevity and valve durability. It is important to emphasize that these patients should not have any anatomical contraindications to transfemoral access and a careful assessment of the balance between the patient's expected longevity and valve life must be taken into consideration. These indications are given predominantly based on large RCTs that have reported favorable outcomes after percutaneous intervention [9, 10, 22, 24–26, 108]. These recommendations have prompted the use of percutaneous intervention even in asymptomatic patients in whom severe AVS was associated with an LVEF < 50% and at an age below 80 years presenting with no anatomical contraindication to the percutaneous transfemoral approach [9, 10, 22, 24–26, 108] (Figure 5).

Concerns related to heart rhythm complications have been clarified. Siontis et al. [109] reported data from a meta-analysis of 7 RCTs and 8020 patients. TAVR had lower risk of strokes (*p* = 0.028) while patients who underwent AVR-S had lower risk of major vascular complications (*p* = 0.001) and permanent pacemaker implantations (*p* < 0.001).

With the advent of the percutaneous interventions, many of the evaluations that were previously made for the choice of the optimal valve type have now faded. We are witnessing a real paradigm shift that concerns both the structural degeneration of the valve and the risk of reoperation as well as the bleeding complications due to the use of anticoagulant therapy.

The consequence has been an increased use of biological valves in the population starting from the age of who can benefit from the percutaneous valve-in-valve procedure after the development of SVD. In this regard, it should be noted that the long-term durability of CEP [100, 101] valve in the aortic position is better than expected in the younger population. In young patients with severe AVS who wish to avoid anticoagulation, the use of bioprosthetic valves is recommended, but strategies aimed at a long-term success of surgery require the longest duration of bioprosthetic valves. The optimal choice of the biological substitute used must be effective with minimum early postoperative gradients to obtain a more effective valve orifice using the appropriate valve size and proceedings with the selective use of the root enlargement. The major concern for the use of transcatheter valve-in-valve procedure as a second intervention for patients who experience SVD is the small-sized prosthesis that was implanted at the initial operation. In this case, the subsequent valve-in-valve placement is not indicated and should be avoided due to the potential risk of early attenuated leaflet motion, transcatheter heart valve thrombosis, and early SVD [84].

Although the results on the risk of SVD are very favorable in patients who have received a TAVR operation compared to those who underwent an AVR-S, these results must be confirmed by randomized multicenter studies that enroll a large number of patients [112–117]. A single RCT NOTION [86] compared the second-generation Hancock bioprostheses included of the CoreValve System with different models of conventional stented xenograft. It is indisputable that the duration of CEP and second-generation Hanchock exceeds that of new bioprostheses such as Trifecta and Mitroflow, noted for the increased reports of early SVD in the follow-up of more than 5 years [102–104].

The use of anticoagulant therapy also extended to bioprostheses, which has been favored by the acquisition of data on the risk of thrombosis using refined 4D CT techniques [64, 69] and pushes towards wider use of biological valves and in particular of the self and expanded devices implanted with a percutaneous approach. The changes in the dynamic anatomy of the root should also be studied alongside the pharmacodynamics of antiplatelet drugs. Concerns regarding variable pharmacodynamic effects of clopidogrel-based dual antiplatelet therapy remain and may lead to the use of more effective antiplatelet agents such as prasugrel or ticagrelor which could provide better antithrombotic effects by avoiding the development of HALT. The findings from ongoing trials such as AUREA (Dual Antiplatelet Therapy Versus Oral Anticoagulation for a Short Time to Prevent Cerebral Embolism After TAVI), ENVISAGE-TAVI AF (Edoxaban Compared to Standard Care After Heart Valve Replacement Using a Catheter in Patients With Atrial Fibrillation), POPular-TAVI (Antiplatelet Therapy for Patients Undergoing Transcatheter Aortic Valve Implantation), and CLOE (Clopidogrel to Lower Adverse Ischemic Events After Transcatheter Aortic Valve) should be integrated with predictive studies on mechanical modeling using computed finite element analysis (FEA) research and 4D CT scan reconstruction. This way, we will not only limit ourselves to antithrombotic treatment optimization but also investigate other variables involved in the thrombotic process.

## 5. Conclusion

THVT and AVR-S are both useful tools in the armamentarium against aortic stenosis. The use of one modality over the other however should be best guided by a strong robust evidence base, ideally with a long-term follow-up. This is best performed by the heart team with the patient as the center of the discussion.

## Figures and Tables

**Table 1:** PRISMA checklist item. RCTs evaluating safety and efficacy of balloon- and self-expanded TAVR. For each study, total population, follow-up, and treatment type are reported. Primary and secondary endpoints are expressed in the number of patients. Main results are summarized in the right column as hazard ratios or incidence rates.

Section and topic	Item #	Table 1 checklist item	Location where item is reported
*Title*			
Title	1	Identify the report as a systematic review.	Title
*Abstract*			
Abstract	2	See the PRISMA 2020 for abstract checklist.	Abstract
*Introduction*			
Rationale	3	Describe the rationale for the review in the context of existing knowledge.	Introduction
Objectives	4	Provide an explicit statement of the objective(s) or question(s) the review addresses.	Introduction
*Methods*			
Eligibility criteria	5	Specify the inclusion and exclusion criteria for the review and how studies were grouped for the syntheses.	Methods
Information sources	6	Specify all databases, registers, websites, organisations, reference lists, and other sources searched or consulted to identify studies. Specify the date when each source was last searched or consulted.	Methods
Search strategy	7	Present the full search strategies for all databases, registers, and websites, including any filters and limits used.	Methods
Selection process	8	Specify the methods used to decide whether a study met the inclusion criteria of the review, including how many reviewers screened each record and each report retrieved, whether they worked independently, and, if applicable, details of automation tools used in the process.	Methods
Data collection process	9	Specify the methods used to collect data from reports, including how many reviewers collected data from each report, whether they worked independently, any processes for obtaining or confirming data from study investigators, and, if applicable, details of automation tools used in the process.	Methods
Data items	10a	List and define all outcomes for which data were sought. Specify whether all results that were compatible with each outcome domain in each study were sought (e.g., for all measures, time points, and analyses) and, if not, the methods used to decide which results to collect.	Methods
	10b	List and define all other variables for which data were sought (e.g., participant and intervention characteristics and funding sources). Describe any assumptions made about any missing or unclear information.	Methods
Study risk of bias assessment	11	Specify the methods used to assess risk of bias in the included studies, including details of the tool(s) used, how many reviewers assessed each study and whether they worked independently, and, if applicable, details of automation tools used in the process.	Methods
Effect measures	12	Specify for each outcome the effect measure(s) (e.g., risk ratio and mean difference) used in the synthesis or presentation of results.	Tables 1 and 2
Synthesis methods	13a	Describe the processes used to decide which studies were eligible for each synthesis (e.g., tabulating the study intervention characteristics and comparing against the planned groups for each synthesis (item #5)).	Methods
	13b	Describe any methods required to prepare the data for presentation or synthesis, such as handling of missing summary statistics, or data conversions.	N/A
	13c	Describe any methods used to tabulate or visually display results of individual studies and syntheses.	Methods
	13d	Describe any methods used to synthesize results and provide a rationale for the choice(s). If meta-analysis was performed, describe the model(s), method(s) to identify the presence and extent of statistical heterogeneity, and software package(s) used.	N/A
	13e	Describe any methods used to explore possible causes of heterogeneity among study results (e.g., subgroup analysis and metaregression).	N/A
	13f	Describe any sensitivity analyses conducted to assess robustness of the synthesized results.	N/A
Reporting bias assessment	14	Describe any methods used to assess risk of bias due to missing results in a synthesis (arising from reporting biases).	Methods
Certainty assessment	15	Describe any methods used to assess certainty (or confidence) in the body of evidence for an outcome.	Tables 1 and 2
*Results*			
Study selection	16a	Describe the results of the search and selection process, from the number of records identified in the search to the number of studies included in the review, ideally using a flow diagram.	Figure 1
	16b	Cite studies that might appear to meet the inclusion criteria, but which were excluded, and explain why they were excluded.	Figure 1
Study characteristics	17	Cite each included study and present its characteristics.	Tables 1 and 2
Risk of bias in studies	18	Present assessments of risk of bias for each included study.	N/A
Results of individual studies	19	For all outcomes, present, for each study (a) summary statistics for each group (where appropriate) and (b) an effect estimate and its precision (e.g., confidence/credible interval), ideally using structured tables or plots.	Tables 1 and 2
Results of syntheses	20a	For each synthesis, briefly summarize the characteristics and risk of bias among contributing studies.	N/A
	20b	Present results of all statistical syntheses conducted. If meta-analysis was done, present for each the summary estimate and its precision (e.g., confidence/credible interval) and measures of statistical heterogeneity. If comparing groups, describe the direction of the effect.	N/A
	20c	Present results of all investigations of possible causes of heterogeneity among study results.	N/A
	20d	Present results of all sensitivity analyses conducted to assess the robustness of the synthesized results.	N/A
Reporting biases	21	Present assessments of risk of bias due to missing results (arising from reporting biases) for each synthesis assessed.	N/A
Certainty of evidence	22	Present assessments of certainty (or confidence) in the body of evidence for each outcome assessed.	Tables 1 and 2
*Discussion*			
Discussion	23a	Provide a general interpretation of the results in the context of other evidence.	Discussion
	23b	Discuss any limitations of the evidence included in the review.	Discussion
	23c	Discuss any limitations of the review processes used.	Discussion
	23d	Discuss implications of the results for practice, policy, and future research.	Discussion
*Other information*			
Registration and protocol	24a	Provide registration information for the review, including register name and registration number, or state that the review was not registered.	N/A
	24b	Indicate where the review protocol can be accessed or state that a protocol was not prepared.	N/A
	24c	Describe and explain any amendments to information provided at registration or in the protocol.	N/A
Support	25	Describe sources of financial or nonfinancial support for the review and the role of the funders or sponsors in the review.	N/A
Competing interests	26	Declare any competing interests of review authors.	Title page
Availability of data, code, and other materials	27	Report which of the following are publicly available and where they can be found: template data collection forms, data extracted from included studies, data used for all analyses, analytic code, and any other materials used in the review.	N/A

Abbreviations. HR = hazard ratio; MEV = mechanically expanding valve; PEP = primary endpoint; RD = risk difference; SEP = secondary endpoint; SEV = self-expanding valve; TAVR = transcatheter heart valve replacement.

**Table 2:** RCTs evaluating safety and efficacy of balloon- and self-expanded TAVR. For each study, total population, follow-up, and treatment type are reported. Primary and secondary endpoints are expressed in the number of patients. The main results are summarized in the right column as hazard ratios or incidence rates. Studies evaluating transcatheter heart valve therapies. An evaluation of THVT and anticoagulant regimen is reported. Studies are summarized according to the main finding in terms of treatment and prevention. The number of total TAVR implantations per study is reported.

Author/year # Type of study	Study completion date	Total number	Treatment (*N*)		Follow-up (yrs)	Primary endpoint (*N*)		Secondary endpoints (*N*)		Finding
Makkar 2020 (23) Lancet RCT	2025	358	TAVR	SAPIEN	2	All-cause mortality or disabling stroke		Severe aortic regurgitation		At 2 yrs, death (*p* = 0.40) and stroke (*p* = 0.23) similar between groups. Portico no advantage
			Portico	CoreValve						
			381	369		Portico	Others	Portico	Others	
						55	48	1/269	0/269	
Lanz 2019 Lancet RCT	2022	739	TAVR SAPIEN 3	TAVR ACURATE neo	30 days	Composite early safety and clinical efficacy		Individual components of primary endpoint, procedural complications, and clinical safety		30 days' safety and clinical efficacy better for SAPIEN 3 vs. ACURATE neo. PEP RD 7.1% (1.3-12.9) *p* = 0.0156
			367	372		SAPIEN 3 60	ACURATE neo 87	SAPIEN 3 255	ACURATE neo 287	
Mack 2019 (11) NEJM RCT	2029	950	TAVR	SAVR	1	Death from any cause, stroke, and rehospitalization at 1 year		Death or stroke at 30 days		At 1 year, the rates of the composite of death, stroke, and rehospitalization lower for TAVR. PEP HR 0.54 (0.37 to 0.79) *p* = 0.001
			496	454		TAVR	SAVR	TAVR	SAVR	
						42	68	5	15	
Popma 2019 (26) NEJM RCT	2026	1468	TAVR	SAVR	2	Composite of death from any cause or stroke		Composite of death, stroke, bleeding, major vascular complication, and others		At 2 yrs, TAVR noninferior with respect to death from any cause or stroke. PEP incidence difference: -1.8 (BCI -4.0-0.4)
			734	734						
						TAVR	SAVR			
						39	49	TAVR 381	SAVR 633	
Thyregod 2019 (27) Circulation RCT	2023	280	TAVR 145	SAVR 135	5	Death from any cause, stroke, or MI		Cardiac death, stroke, TIA, MI, AF, pacemaker, reintervention, and endocarditis		Higher rates of prosthetic regurgitation and pacemaker implantation higher for TAVR PEP, *p* = 0.86
						TAVR 55	SAVR 49			
								TAVR 167	SAVR 155	
Feldman, 2018 (28) JAMA RCT	2025	912	TAVR MEV 607	TAVR SEV 305	1	Composite of all-cause mortality, stroke, and moderate or greater paravalvular leak noninferiority		1-year moderate or greater paravalvular leak, superiority testing		Higher rates of new pacemaker implants and valve thrombosis higher for MEV. Higher rates of repeat procedures, valve-in-valve and valve malpositioning for SEV. PEP *p* < 0.001 for noninferiority
								MEV 5	SEV 21	
						MEV 93	SEV 78			
Reardon 2017 (25) NEJM RCT	2026	1660	TAVR 864	SAVR 796	2	Death from any cause and stroke		MACE and cerebrovascular events		SAVR higher acute kidney injury, AF, and blood transfusion. TAVR higher rates of residual AR and PM implantation. PEP 95% credible interval posterior probability of noninferiority > 0.999
						TAVR 109	SAVR 111	—	—	
Leon 2016 (35) NEJM RCT	2024	2032	TAVR 1011	SAVR 1021	2	Death from any cause or disabling stroke		Death, stroke, or rehospitalization		TAVR similar to SAVR for primary endpoint of death or disabling stroke. PEP HR 0.89 (0.73 to 1.09) *p* = 0.25
						TAVR 192	SAVR 202	TAVR 344	SAVR 326	
Mack 2015 (10) Lancet RCT	2017	699	TAVR 348	SAVR 351	5	Death from any cause		Death or stroke at 5 yrs		TAVR similar clinical outcomes in high surgical risk patients. PEP HR 1.04, 95% CI 0.86–1.24; *p* = 0.76
						TAVR 229	SAVR 198	TAVR 236	SAVR 200	
Kapadia 2015 (9) Lancet RCT	2017	358	TAVR 179	SAVR 179	5	Death from any cause		Cardiovascular death, stroke, vascular complications, major bleeding, and functional status		At 2 yrs rates of cardiovascular mortality lower for TAVR. PEP HR 0.50, 95% CI 0.39–0.65; *p* < 0.0001
						TAVR 127	SAVR 143	TAVR 84	SAVR 118	
Adams 2014 (24) NEJM RCT	2019	795	TAVR 394	SAVR 401	1	Rate of death from any cause at 1 year		Composite of death from any cause, myocardial infarction, stroke, and reintervention		At 1 yr. survival higher for TAVR. Adverse event more frequent for TAVR. PEP *p* < 0.001 for noninferiority
						TAVR 14.2%	SAVR 19.1%	TAVR 90	SAVR 211	
Kodali 2012 (96) NEJM RCT	2017	699	TAVR 348	SAVR 351	2	Death from any cause		Cardiovascular mortality, stroke, rehospitalization, AKI, vascular complications, bleeding, and NYHA		PVL higher for TAVR and associated with increased late mortality. PEP HR 0.90; 95% CI, 0.71 to 1.15; *p* = 0.41
						TAVR 116	SAVR 114	TAVR 295	SAVR 266	
Makkar 2012 (3) NEJM RCT	2017	358	TAVR 179	MT 179	2	Death from any cause		Death or stroke at 2 yrs		At 2 years, TAVR reduced the rates of death and hospitalization. PEP HR 0.95 (0.91 to 0.98); *p* = 0.005
						TAVR 77	OMT 117	TAVR 82	OMT 117	

Abbreviations: HR = hazard ratio; MEV = mechanically expanding valve; PEP = primary endpoint; RD = risk difference; SEP = secondary endpoint; SEV = self-expanding valve; TAVR = transcatheter heart valve replacement; HALT = hypoattenuated leaflet thickening; NOACs = new oral anticoagulants; PR = platelet reactivity; THVT = transcatheter heart valve thrombosis; VKA = vitamin K antagonists.

**Table 3:** Studies evaluating THVT. Studies evaluating transcatheter heart valve therapies. An evaluation of THVT and anticoagulant regimen is reported. Studies are summarized according to the main finding in terms of treatment and prevention. The number of total TAVR implantations per study is reported. Studies reporting structural valve deterioration of bioprosthetic valves. SVD estimates for each study. Reoperation for SVD, freedom from SVD, and freedom from cardiac mortality are some of the reported results. For each study, population samples and number of implanted bioprosthesis are depicted.

First author/type of study (Ref. #)	Total sample (*N*)	Mean follow-up (months)	Number of TAVR implanted (actually evaluated)	Main findings
De Backer 2019 (52) NEJM RCT	231	3	231 (198)	Less subclinical leaflet motion abnormalities with rivaroxaban compared to antiplatelet but higher death risk.
Nührenberg 2019 (51) JACC CardioInt OS	331	5 days	331 (200)	ADP-induced PR not associated with HALT. Its incidence was decreased by oral anticoagulants.
Collet 2018 (53) AHJ RCT	1510 planned	12	1510 planned	Superiority of apixaban vs. standard VKA or antiplatelet to reduce post-TAVR thromboembolism and bleeding is tested.
Chakravarty 2017 (48) Lancet OS	931	19	752	Subclinical leaflet thrombosis more frequent with TAVR than SAVR. NOACs and warfarin more effective in treating and preventing it.
Hansson 2016 (43) JACC OS	460	3	460	Larger THV size associated with THV thrombosis. Warfarin showed a protective effect.
Vollema 2016 (49) EHJ OS	434	36	434 (128)	12.5% showed HALT or reduced leaflet motion. Neither HALT nor increased transvalvular gradient associated with stroke or TIA.
Pache 2015 (50) EHJ OS	249	5 days	249 (156)	Early HALT did not present with symptoms. Not associated with antiplatelet regimen type. Reversible with VKAs.
Makkar 2015 (47) NEJM RCT/registry	187	6	160	Reduced leaflet motion reversed by anticoagulation. No differences in stroke or TIA with respect to normal leaflet motion.

Abbreviations. HALT = hypoattenuated leaflet thickening; NOACs = new oral anticoagulants; PR = platelet reactivity; THVT = transcatheter heart valve thrombosis; VKA = vitamin K antagonists; CEP = Carpentier-Edwards Perimount; OPM = observation propensity matched; OS = observation study; RCT = randomized clinical trial; SVD = structural valve deterioration; SFS = Sorin Freedom Solo; MST = median survival time.

**Table 4:** Studies reporting structural valve deterioration of bioprosthetic valves. SVD estimates for each study. Reoperation for SVD, freedom from SVD, and freedom from cardiac mortality are some of the reported results. For each study, population samples and number of implanted bioprosthesis are depicted.

First author/type of study (Ref. #)	Total sample (*N*)	Mean follow-up (months)	Number of bioprostheses implanted	Main findings
Schaefer 2018 PLoS One/OPM (79)	#154	48.7	SFS (77)	SVD (5.2% SFS vs. 0% CP; *p* = 0.04). Reoperation for SVD (9.1% SFS vs. 1.3% CP; *p* = 0.04)
			CEP bioprosthesis (77)	
Goldman 2017 JTCVS (87)	710	72	Trifecta bioprosthesis	6 y freedom from mortality and paravalvular leak 98.3% and 98.9%, respectively. 6 y freedom from reoperation for SVD 95.7%
Wang 2017 Ann Thorac Surg/MA (73)	42.305	Mean times to valve failure (MTTF)	Medtronic porcine (9.619)	Higher SVD risk for Sorin. No significant differences in SVD risk for other three valve types (*p* = 0.716)
			Edwards porcine (3.886)	
			Sorin pericardial (6.632)	
			CEP bioprosthesis (22.177)	
Goldstone 2017 NEJM/OPM ()	9942	60	Xenograft (3845)	Higher incidence of reoperation for biologic prosthesis
				Hazard ratio 2.60 (95% CI 1.91 to 3.40) patients from 45 to 55
				Hazard ratio 2.46 (95% CI 1.93 to 3.20) patients from 55 to 64
Fischlein 2016 JTCVS (89)	658	12	LivaNova Perceval Sutureless	1 y freedom from cardiac death 95.4%. No valve thrombosis, no SVD reported. 1 y paravalvular leak incidence 1.1%
Foroutan 2016 BMJ/MA (74)	53,884	Cumulative incidence of death and SVD at 10, 15, and 20 yrs	53,884	Freedom from SVD 94.0%, 81.7%, and 52% at 10, 15, and 20 yrs
				The rate of SVD increases rapidly after 10 yrs and particularly after 15 yrs
Bourguignon 2015 Ann Thorac Surg/OS (84)	2559	79	XP model (111) CEP bioprosthesis (others)	20 yr freedom from SVD 48.5%. MST 19.7 years (95% CI 18.5% to 21.1%). Cumulative risk of reoperation for SVD HR 0.93 (95% CI 0.92 to 0.94; *p* < 0.001)
Johnston 2015 OS Ann Thorac Surg/OS (85)	12,569	68	XP (450)	Few probabilities of explantation for SVD (5.4%) at 20 years
			CEP bioprosthesis (others)	
Chiang 2014 JAMA/OPM	1001	127.2	XP (500)	The cumulative incidence for bioprosthesis reoperation
				12.1% (95% CI, 8.8%-15.4%) at 15 years
Sénage 2014 Circulation/OPM (86)	617	44	Mitroflow (models 12A/LX)	Early SVD. 1-, 2-, and 5-year 0.2% (95% confidence interval (CI), 0.0–0.6), 0.8% (95% CI, 0.0–1.6), and 8.4% (95% CI, 5.3–11.3). 5-year freedom from SVD 91.6% (95% confidence interval (CI), 88.7–94.7). 13 patients accelerated SVD
Glaser 2014 Ann Thorac Surg/OPM (83)	1219	50	CEP (864)	No difference in reoperation for SVD (*p* = 0.745)
			Hancock second generation (365)	
Amabile 2014 JTCVS/OS (90)	500	104.8	Freestyle bioprosthesis (Medtronic Inc, Minneapolis, Minn)	Freedom from SVD 94% at 10 yrs (0.6% per patient/yr.). Aged less than 65 yrs freedom from SVD 89% at 10 yrs
Kocher 2013 JTCVS/RCT	152	9.8	EDWARDS INTUITY Valve System	Valve-related mortality 1.4%. Early and late paravalvular leak 1.4% and 0.9%. Early valve-related pacemaker 5%
Garrido-Olivares 2011 Ann Thorac Surg/OS (81)	#1076	166	Hancock II Bioprosthesis 1076	Reoperation for SVD 68.5% at 20 yrs
David 2008 JTCVS/OS (78)	357	91	SPV (T-SPV) bioprosthesis (St Jude Medical, Inc, St Paul, Minn)	Freedom from SVD 69% at 12 yrs (52% for patients less than 65 years of age and 85% for patients 65 yrs of age or older) (*p* = 0.002)

Abbreviations: CEP = Carpentier-Edwards Perimount; OPM = observation propensity matched; OS = observation study; RCT = randomized clinical trial; SVD = structural valve deterioration; SFS = Sorin Freedom Solo; MST = median survival time.

## References

[1] Otto C. M., Prendergast B. (2014). Aortic-valve stenosis--from patients at risk to severe valve obstruction. *The New England Journal of Medicine*.

[2] Eveborn G. W., Schirmer H., Heggelund G., Lunde P., Rasmussen K. (2013). The evolving epidemiology of valvular aortic stenosis: the Tromsø Study. *Heart*.

[3] Makkar R. R., Fontana G. P., Jilaihawi H. (2012). Transcatheter aortic-valve replacement for inoperable severe aortic stenosis. *The New England Journal of Medicine*.

[4] Kodali S. K., Williams M. R., Smith C. R. (2012). Two-year outcomes after transcatheter or surgical aortic-valve replacement. *The New England Journal of Medicine*.

[5] (2010). *National Hospital Discharge Survey: number of all listed procedures for discharges from short-stay hospitals, by ICD- 9-CM code, sex, age, and geographic region: United States, 2010*.

[6] Leon M. B., Smith C. R., Mack M. (2010). Transcatheter aortic-valve implantation for aortic stenosis in patients who cannot undergo surgery. *The New England Journal of Medicine*.

[7] Otto C. M., Nishimura R. A., Bonow R. O. (2021). 2020 ACC/AHA guideline for the management of patients with valvular heart disease: executive summary: a report of the American College of Cardiology/American Heart Association joint committee on clinical practice guidelines. *Circulation*.

[8] Zhang X. L., Zhang X. W., Lan R. F. (2021). Long-term and temporal outcomes of transcatheter versus surgical aortic-valve replacement in severe aortic stenosis: a meta-analysis. *Annals of Surgery*.

[9] Mack M. J., Leon M. B., Smith C. R. (2015). 5-year outcomes of transcatheter aortic valve replacement or surgical aortic valve replacement for high surgical risk patients with aortic stenosis (PARTNER 1): a randomised controlled trial. *Lancet*.

[10] Mack M. J., Leon M. B., Thourani V. H. (2019). Transcatheter aortic-valve replacement with a balloon-expandable valve in low-risk patients. *The New England Journal of Medicine*.

[11] Vahanian A., Alfieri O., Andreotti F. (2012). Guidelines on the management of valvular heart disease (version 2012). *European Heart Journal*.

[12] Falk V. (2014). Transcatheter aortic valve replacement indications should not be expanded to lower-risk and younger patients. *Circulation*.

[13] Kapadia S. R., Leon M. B., Makkar R. R. (2015). 5-year outcomes of transcatheter aortic valve replacement compared with standard treatment for patients with inoperable aortic stenosis (PARTNER 1): a randomised controlled trial. *The Lancet*.

[14] Michelena H. I., Desjardins V. A., Avierinos J.-F. (2008). Natural history of asymptomatic patients with normally functioning or minimally dysfunctional bicuspid aortic valve in the community. *Circulation*.

[15] Siu S. C., Silversides C. K. (2010). Bicuspid aortic valve disease. *Journal of the American College of Cardiology*.

[16] Braverman A. C., Otto C. M., Bonow R. O. (2013). The bicuspid aortic valve and associated aortic disease. *Valvular Heart Disease*.

[17] Webb J. G., Altwegg L., Boone R. H. (2009). Transcatheter aortic valve implantation: impact on clinical and valve-related outcomes. *Circulation*.

[18] Nishimura R. A., Otto C. M., Bonow R. O. (2014). 2014 AHA/ACC guideline for the management of patients with valvular heart disease: a report of the American College of Cardiology/American Heart Association Task Force on Practice Guidelines. *Journal of the American College of Cardiology*.

[19] Nishimura R. A., Otto C. M., Bonow R. O. (2017). 2017 AHA/ACC focused update of the 2014 AHA/ACC guideline for the management of patients with valvular heart disease. *Journal of the American College of Cardiology*.

[20] Baumgartner H., Falk V., Bax J. J. (2017). 2017 ESC/EACTS guidelines for the management of valvular heart disease. *European Heart Journal*.

[21] Smith C. R., Leon M. B., Mack M. J. (2011). Transcatheter versus surgical aortic-valve replacement in high-risk patients. *The New England Journal of Medicine*.

[22] Makkar R. R., Thourani V. H., Mack M. J. (2020). Five-year outcomes of transcatheter or surgical aortic-valve replacement. *The New England Journal of Medicine*.

[23] Makkar R. R., Cheng W., Waksman R. (2020). Self-expanding intra-annular versus commercially available transcatheter heart valves in high and extreme risk patients with severe aortic stenosis (PORTICO IDE): a randomised, controlled, non-inferiority trial. *The Lancet*.

[24] Adams D. H., Popma J. J., Reardon M. J. (2014). Transcatheter aortic-valve replacement with a self-expanding prosthesis. *The New England Journal of Medicine*.

[25] Reardon M. J., Van Mieghem N. M., Popma J. J. (2017). Surgical or transcatheter aortic-valve replacement in intermediate-risk patients. *The New England Journal of Medicine*.

[26] Popma J. J., Deeb G. M., Yakubov S. J. (2019). Transcatheter aortic-valve replacement with a self-expanding valve in low-risk patients. *The New England Journal of Medicine*.

[27] Thyregod H. G. H., Ihlemann N., Jørgensen T. H. (2019). Five-year clinical and echocardiographic outcomes from the NOTION randomized clinical trial in Patients at lower surgical risk. *Circulation*.

[28] Feldman T. E., Reardon M. J., Rajagopal V. (2018). Effect of mechanically expanded vs self-expanding transcatheter aortic valve replacement on mortality and major adverse clinical events in high-risk patients with aortic stenosis: the REPRISE III randomized clinical trial. *JAMA*.

[29] Walther T., Simon P., Dewey T. (2007). Transapical minimally invasive aortic valve implantation: multicenter experience. *Circulation*.

[30] Grube E., Schuler G., Buellesfeld L. (2007). Percutaneous Aortic Valve Replacement for Severe Aortic Stenosis in High-Risk Patients Using the Second- and Current Third-Generation Self-Expanding CoreValve Prosthesis: Device Success and 30-Day Clinical Outcome. *Journal of the American College of Cardiology*.

[31] Varadarajan P., Kapoor N., Bansal R. C., Pai R. G. (2006). Survival in elderly patients with severe aortic stenosis is dramatically improved by aortic valve replacement: results from a cohort of 277 patients aged ≥80 years. *European Journal of Cardio-Thoracic Surgery*.

[32] Cribier A., Eltchaninoff H., Tron C. (2006). Treatment of Calcific Aortic Stenosis With the Percutaneous Heart Valve: Mid- Term Follow-Up From the Initial Feasibility Studies: The French Experience. *Journal of the American College of Cardiology*.

[33] Rodés-Cabau J., Webb J. G., Cheung A. (2010). Transcatheter aortic valve implantation for the treatment of severe symptomatic aortic stenosis in patients at very high or prohibitive surgical risk. *Journal of the American College of Cardiology*.

[34] Tamburino C., Capodanno D., Ramondo A. (2011). Incidence and predictors of early and late mortality after transcatheter aortic valve implantation in 663 patients with severe aortic stenosis. *Circulation*.

[35] Leon M. B., Smith C. R., Mack M. J. (2016). Transcatheter or surgical aortic-valve replacement in intermediate-risk patients. *The New England Journal of Medicine*.

[36] Deeb G. M., Reardon M. J., Chetcuti S. (2016). 3-Year outcomes in high-risk patients who underwent surgical or transcatheter aortic valve replacement. *Journal of the American College of Cardiology*.

[37] O'Brien S. M., Shahian D. M., Filardo G. (2009). The Society of Thoracic Surgeons 2008 Cardiac Surgery Risk Models: Part 2-- Isolated Valve Surgery. *The Annals of Thoracic Surgery*.

[38] Green P., Woglom A. E., Genereux P. (2012). The Impact of Frailty Status on Survival After Transcatheter Aortic Valve Replacement in Older Adults With Severe Aortic Stenosis: A Single-Center Experience. *JACC. Cardiovascular Interventions*.

[39] Reardon M. J., Adams D. H., Kleiman N. S. (2015). 2-year outcomes in patients undergoing surgical or self-expanding transcatheter aortic valve replacement. *Journal of the American College of Cardiology*.

[40] Latib A., Naganuma T., Abdel-Wahab M. (2015). Treatment and clinical outcomes of transcatheter heart valve thrombosis. *Circulation. Cardiovascular Interventions*.

[41] Stortecky S., Windecker S. (2012). Stroke: an infrequent but devastating complication in cardiovascular interventions. *Circulation*.

[42] Leetmaa T., Hansson N. C., Leipsic J. (2015). Early aortic transcatheter heart valve thrombosis: diagnostic value of contrast-enhanced multidetector computed tomography. *Circulation. Cardiovascular Interventions*.

[43] Hansson N. C., Grove E. L., Andersen H. R. (2016). Transcatheter aortic valve thrombosis. *Journal of the American College of Cardiology*.

[44] Wolberg A. S., Aleman M. M., Leiderman K., Machlus K. R. (2012). Procoagulant activity in hemostasis and thrombosis: Virchow’s triad revisited. *Anesthesia and Analgesia*.

[45] Turbill P., Beugeling T., Poot A. A. (1996). Proteins involved in the Vroman effect during exposure of human blood plasma to glass and polyethylene. *Biomaterials*.

[46] Noble S., Asgar A., Cartier R., Virmani R., Bonan R. (2009). Anatomo-pathological analysis after CoreValve Revalving system implantation. *EuroIntervention*.

[47] Makkar R. R., Fontana G., Jilaihawi H. (2015). Possible subclinical leaflet thrombosis in bioprosthetic aortic valves. *The New England Journal of Medicine*.

[48] Chakravarty T., Søndergaard L., Friedman J. (2017). Subclinical leaflet thrombosis in surgical and transcatheter bioprosthetic aortic valves: an observational study. *Lancet*.

[49] Vollema E. M., Kong W. K. F., Katsanos S. (2017). Transcatheter aortic valve thrombosis: the relation between hypo-attenuated leaflet thickening, abnormal valve haemodynamics, and stroke. *European Heart Journal*.

[50] Pache G., Schoechlin S., Blanke P. (2016). Early hypo-attenuated leaflet thickening in balloon expandable transcatheter aortic heart valves. *European Heart Journal*.

[51] Nührenberg T. G., Hromek J., Kille A. (2019). Impact of on-clopidogrel platelet reactivity on incidence of hypoattenuated leaflet thickening after transcatheter aortic valve replacement. *JACC. Cardiovascular Interventions*.

[52] de Backer O., Dangas G. D., Jilaihawi H. (2020). Reduced leaflet motion after transcatheter aortic-valve replacement. *The New England Journal of Medicine*.

[53] Collet J. P., Berti S., Cequier A. (2018). Oral anti-Xa anticoagulation after trans-aortic valve implantation for aortic stenosis: The randomized ATLANTIS trial. *American Heart Journal*.

[54] Nappi F., Mazzocchi L., Avtaar Singh S. S. (2018). Complementary role of the computed biomodelling through finite element analysis and computed tomography for diagnosis of transcatheter heart valve thrombosis. *BioMed Research International*.

[55] Morganti S., Conti M., Aiello M. (2014). Simulation of transcatheter aortic valve implantation through patient-specific finite element analysis: Two clinical cases. *Journal of Biomechanics*.

[56] Morganti S., Brambilla N., Petronio A. S., Reali A., Bedogni F., Auricchio F. (2016). Prediction of patient-specific post-operative outcomes of TAVI procedure: The impact of the positioning strategy on valve performance. *Journal of Biomechanics*.

[57] Bonhoeffer P., Boudjemline Y., Saliba Z. (2000). Transcatheter implantation of a bovine valve in pulmonary position: a lamb study. *Circulation*.

[58] Vesely I., Casarotto D. C., Gerosa G. (2000). Mechanics of cryopreserved aortic and pulmonary homografts. *The Journal of Heart Valve Disease*.

[59] Spadaccio C., Mazzocchi L., Timofeva I. (2020). Bioengineering case study to evaluate complications of adverse anatomy of aortic root in transcatheter aortic valve replacement: combining biomechanical modelling with CT imaging. *Bioengineering (Basel).*.

[60] Nappi F., Attias D., Avtaar Singh S. S., Prot V. (2019). Finite element analysis applied to the transcatheter mitral valve therapy: Studying the present, imagining the future. *The Journal of Thoracic and Cardiovascular Surgery*.

[61] Nappi F., Mazzocchi L., Timofeva I. (2020). A finite element analysis study from 3D CT to predict transcatheter heart valve thrombosis. *Diagnostics (Basel).*.

[62] Spadaccio C., Fraldi M., Sablayrolles J. L., Nappi F. J. (2016). TAVI in lower risk patients: revolution or nonsense? Keep calm and select patients. *American College of Cardiology*.

[63] Nappi F., Spadaccio C., Sablayrolles J. L. (2016). Pushing the limits in transcatheter aortic valve replacement: high-volume center's effect, overconfidence, or something else?. *JACC. Cardiovascular Interventions*.

[64] Nappi F., Nenna A., Larobina D. (2018). Simulating the ideal geometrical and biomechanical parameters of the pulmonary autograft to prevent failure in the Ross operation. *Interactive Cardiovascular and Thoracic Surgery*.

[65] Nappi F., Spadaccio C., Sablayrolles J. L. (2017). Delayed prosthesis malposition after transcatheter aortic valve implantation causing coronaries obstruction. *European Journal of Cardio-Thoracic Surgery*.

[66] Spadaccio C., Mozetic P. (2016). Cells and extracellular matrix interplay in cardiac valve disease: because age matters. *Basic Research in Cardiology*.

[67] Nappi F., Spadaccio C., Al-Attar N., Acar C. (2015). The Ross procedure at the crossroads: Lessons from biology: Is Dr Ross's dream concluded?. *International Journal of Cardiology*.

[68] Spadaccio C., Montagnani S., Acar C., Nappi F. (2015). Introducing bioresorbable scaffolds into the show. A potential adjunct to resuscitate Ross procedure. *International Journal of Cardiology*.

[69] Spadaccio C., Nappi F., de Marco F. (2016). Preliminary in vivo evaluation of a hybrid armored vascular graft combining electrospinning and additive manufacturing techniques. *Drug Target Insights*.

[70] Spadaccio C., Rainer A., Mozetic P. (2015). The role of extracellular matrix in age-related conduction disorders: a forgotten player?. *Journal of Geriatric Cardiology*.

[71] Nappi F., Spadaccio C., Fraldi M. (2016). A composite semiresorbable armoured scaffold stabilizes pulmonary autograft after the Ross operation: Mr Ross's dream fulfilled. *The Journal of Thoracic and Cardiovascular Surgery*.

[72] Nappi F., Spadaccio C., Castaldo C. (2014). Reinforcement of the pulmonary artery autograft with a polyglactin and polydioxanone mesh in the Ross operation: experimental study in growing lamb. *The Journal of Heart Valve Disease*.

[73] Nappi F., Carotenuto A. R., Cutolo A. (2016). Compliance mismatch and compressive wall stresses drive anomalous remodelling of pulmonary trunks reinforced with Dacron grafts. *Journal of the Mechanical Behavior of Biomedical Materials*.

[74] Spadaccio C., Nappi F., de Marco F. (2017). Implantation of a poly-L-lactide GCSF-functionalized scaffold in a model of chronic myocardial infarction. *Journal of Cardiovascular Translational Research*.

[75] Nappi F., Fraldi M., Spadaccio C. (2016). Biomechanics drive histological wall remodeling of neoaortic root: a mathematical model to study the expression levels of ki 67, metalloprotease, and apoptosis transition. *Journal of Biomedical Materials Research. Part A*.

[76] Whitlock R. P., Sun J. C., Fremes S. E., Rubens F. D., Teoh K. H. (2012). Antithrombotic and thrombolytic therapy for valvular disease: antithrombotic therapy and prevention of thrombosis, 9th ed: American College of Chest Physicians Evidence-Based Clinical Practice Guidelines. *Chest*.

[77] Brennan J. M., Edwards F. H., Zhao Y. (2012). Early anticoagulation of bioprosthetic aortic valves in older patients. *Journal of the American College of Cardiology*.

[78] Mérie C., Køber L., Skov Olsen P. (2012). Association of warfarin therapy duration after bioprosthetic aortic valve replacement with risk of mortality, thromboembolic complications, and bleeding. *JAMA*.

[79] Cheng D., Pepper J., Martin J. (2009). Stentless versus stented bioprosthetic aortic Valves. *Innovations (Phila)*.

[80] Zoghbi W. A., Chambers J. B., Dumesnil J. G. (2009). Recommendations for evaluation of prosthetic valves with echocardiography and doppler ultrasound: a report From the American Society of Echocardiography’s Guidelines and Standards Committee and the Task Force on Prosthetic Valves, developed in conjunction with the American College of Cardiology Cardiovascular Imaging Committee, Cardiac Imaging Committee of the American Heart Association, the European Association of Echocardiography, a registered branch of the European Society of Cardiology, the Japanese Society of Echocardiography and the Canadian Society of Echocardiography, endorsed by the American College of Cardiology Foundation, American Heart Association, European Association of Echocardiography, a registered branch of the European Society of Cardiology, the Japanese Society of Echocardiography, and Canadian Society of Echocardiography. *Journal of the American Society of Echocardiography*.

[81] Kappetein A. P., Head S. J., Généreux P. (2012). Updated Standardized Endpoint Definitions for Transcatheter Aortic Valve Implantation:. *Journal of the American College of Cardiology*.

[82] Lancellotti P., Pibarot P., Chambers J. (2016). Recommendations for the imaging assessment of prosthetic heart valves: a report from the European Association of Cardiovascular Imaging endorsed by the Chinese Society of Echocardiography, the Inter-American Society of Echocardiography, and the Brazilian Department of Cardiovascular Imaging. *European Heart Journal Cardiovascular Imaging*.

[83] Capodanno D., Petronio A. S., Prendergast B. (2017). Standardized definitions of structural deterioration and valve failure in assessing long-term durability of transcatheter and surgical aortic bioprosthetic valves: a consensus statement from the European Association of Percutaneous Cardiovascular Interventions (EAPCI) endorsed by the European Society of Cardiology (ESC) and the European Association for Cardio-Thoracic Surgery (EACTS). *European Journal of Cardio-Thoracic Surgery*.

[84] Attias D., Nejjari M., Nappi F. (2018). How to treat severe symptomatic structural valve deterioration of aortic surgical bioprosthesis: transcatheter valve-in-valve implantation or redo valve surgery?.

[85] Zilberszac R., Gabriel H., Schemper M. (2013). Outcome of combined stenotic and regurgitant aortic valve disease. *Journal of the American College of Cardiology*.

[86] Søndergaard L., Ihlemann N., Capodanno D. (2019). Durability of transcatheter and surgical bioprosthetic aortic valves in patients at lower surgical risk. *Journal of the American College of Cardiology*.

[87] Masters R. G., Walley V. M., Pipe A. L., Keon W. J. (1995). Long-term experience with the Ionescu-Shiley pericardial valve. *The Annals of Thoracic Surgery*.

[88] Puvimanasinghe J. P., Steyerberg E. W., Takkenberg J. J. (2001). Prognosis after aortic valve replacement with a bioprosthesis: predictions based on meta-analysis and microsimulation. *Circulation*.

[89] Wang M., Furnary A. P., Li H. F., Grunkemeier G. L. (2017). Bioprosthetic Aortic Valve Durability: A Meta-Regression of Published Studies. *The Annals of Thoracic Surgery*.

[90] Foroutan F., Guyatt G. H., O'Brien K. (2016). Prognosis after surgical replacement with a bioprosthetic aortic valve in patients with severe symptomatic aortic stenosis: systematic review of observational studies. *BMJ*.

[91] Nappi F., Nenna A., Petitti T. (2018). Long-term outcome of cryopreserved allograft for aortic valve replacement. *The Journal of Thoracic and Cardiovascular Surgery*.

[92] Fukushima S., Tesar P. J., Pearse B. (2014). Long-term clinical outcomes after aortic valve replacement using cryopreserved aortic allograft. *The Journal of Thoracic and Cardiovascular Surgery*.

[93] Arabkhani B., Bekkers J. A., Andrinopoulou E. R., Roos-Hesselink J. W., Takkenberg J. J. M., Bogers A. J. J. C. (2016). Allografts in aortic position: Insights from a 27-year, single-center prospective study. *The Journal of Thoracic and Cardiovascular Surgery*.

[94] David T. E., Feindel C. M., Bos J., Ivanov J., Armstrong S. (2008). Aortic valve replacement with Toronto SPV bioprosthesis: Optimal patient survival but suboptimal valve durability. *The Journal of Thoracic and Cardiovascular Surgery*.

[95] Schaefer A., Dickow J., Schoen G. (2018). Stentless vs stented bioprosthesis for aortic valve replacement: a case matched comparison of long-term follow-up and subgroup analysis of patients with native valve endocarditis. *PLoS One*.

[96] Nishida T., Tominaga R. (2013). A look at recent improvements in the durability of tissue valves. *General Thoracic and Cardiovascular Surgery*.

[97] Garrido-Olivares L., Maganti M., Armstrong S., David T. (2011). Aortic valve replacement with Hancock II bioprothesis with and without replacement of the ascending aorta. *The Annals of Thoracic Surgery*.

[98] David T. E., Armstrong S., Maganti M. (2010). Hancock II bioprosthesis for aortic valve replacement: the gold standard of bioprosthetic valves durability?. *The Annals of Thoracic Surgery*.

[99] Glaser N., Franco-Cereceda A., Sartipy U. (2014). Late Survival After Aortic Valve Replacement With the Perimount Versus the Mosaic Bioprosthesis. *The Annals of Thoracic Surgery*.

[100] Bourguignon T., Bouquiaux-Stablo A. L., Candolfi P. (2015). Very long-term outcomes of the Carpentier-Edwards Perimount valve in aortic position. *The Annals of Thoracic Surgery*.

[101] Johnston D. R., Soltesz E. G., Vakil N. (2015). Long-Term Durability of Bioprosthetic Aortic Valves: Implications From 12,569 Implants. *The Annals of Thoracic Surgery*.

[102] Sénage T., le Tourneau T., Foucher Y. (2014). Early structural valve deterioration of Mitroflow aortic bioprosthesis: mode, incidence, and impact on outcome in a large cohort of patients. *Circulation*.

[103] Goldman S., Cheung A., Bavaria J. E., Petracek M. R., Groh M. A., Schaff H. V. (2017). Midterm, multicenter clinical and hemodynamic results for the Trifecta aortic pericardial valve. *The Journal of Thoracic and Cardiovascular Surgery*.

[104] Kalra A., Rehman H., Ramchandani M. (2017). Early Trifecta valve failure: Report of a cluster of cases from a tertiary care referral center. *The Journal of Thoracic and Cardiovascular Surgery*.

[105] Fischlein T., Meuris B., Hakim-Meibodi K. (2016). The sutureless aortic valve at 1 year: A large multicenter cohort study. *The Journal of Thoracic and Cardiovascular Surgery*.

[106] Kocher A. A., Laufer G., Haverich A. (2013). One-year outcomes of the Surgical Treatment of Aortic Stenosis With a Next Generation Surgical Aortic Valve (TRITON) trial: A prospective multicenter study of rapid-deployment aortic valve replacement with the EDWARDS INTUITY Valve System. *The Journal of Thoracic and Cardiovascular Surgery*.

[107] Durand E., Tron C., Eltchaninoff H. (2015). Emergency transcatheter aortic valve implantation for acute and early failure of sutureless Perceval aortic valve. *The Canadian Journal of Cardiology*.

[108] Siemieniuk R. A., Agoritsas T., Manja V. (2016). Transcatheter versus surgical aortic valve replacement in patients with severe aortic stenosis at low and intermediate risk: systematic review and meta-analysis. *BMJ*.

[109] Siontis G. C. M., Overtchouk P., Cahill T. J. (2019). Transcatheter aortic valve implantation vs. surgical aortic valve replacement for treatment of symptomatic severe aortic stenosis: an updated meta-analysis. *European Heart Journal*.

[110] Siontis K. C., Killu A. M. (2019). Silent and non-silent thromboembolic events after ventricular tachycardia ablation: modifiable risk with postprocedure anticoagulation?. *Journal of Cardiovascular Electrophysiology*.

[111] Thourani V. H., Kodali S., Makkar R. R. (2016). Transcatheter aortic valve replacement versus surgical valve replacement in intermediate-risk patients: a propensity score analysis. *Lancet*.

[112] Barbanti M., Costa G., Zappulla P. (2018). Incidence of long-term structural valve dysfunction and bioprosthetic valve failure after transcatheter aortic valve replacement. *Journal of the American Heart Association*.

[113] Didier R., Eltchaninoff H., Donzeau-Gouge P. (2018). Five-year clinical outcome and valve durability after transcatheter aortic valve replacement in high-risk patients. *Circulation*.

[114] Panico R. A., Giannini C., de Carlo M. (2019). Long-term results and durability of the CoreValve transcatheter aortic bioprosthesis: outcomes beyond five years. *EuroIntervention*.

[115] Durand E., Sokoloff A., Urena-Alcazar M. (2019). Assessment of long-term structural deterioration of transcatheter aortic bioprosthetic valves using the new European definition. *Circulation. Cardiovascular Interventions*.

[116] Chiang Y. P., Chikwe J., Moskowitz A. J., Itagaki S., Adams D. H., Egorova N. N. (2014). Survival and long-term outcomes following bioprosthetic vs mechanical aortic valve replacement in patients aged 50 to 69 years. *Journal of the American Medical Association*.

[117] Goldstone A. B., Chiu P., Baiocchi M. (2017). Mechanical or biologic prostheses for aortic-valve and mitral-valve replacement. *The New England Journal of Medicine*.

[118] Otto C. M., Pearlman A. S. (1988). Doppler echocardiography in adults with symptomatic aortic stenosis: diagnostic utility and cost-effectiveness. *Archives of Internal Medicine*.

[119] TURINA J., HESS O., SEPULCRI F., KRAYENBUEHL H. P. (1987). Spontaneous course of aortic valve disease. *European Heart Journal*.

[120] Kelly T. A., Rothbart R. M., Cooper C. M., Kaiser D. L., Smucker M. L., Gibson R. S. (1988). Comparison of outcome of asymptomatic to symptomatic patients older than 20 years of age with valvular aortic stenosis. *The American Journal of Cardiology*.

[121] Pellikka P. A., Nishimura R. A., Bailey K. R., Tajik A. J. (1990). The natural history of adults with asymptomatic, hemodynamically significant aortic stenosis. *Journal of the American College of Cardiology*.

[122] Dahl J. S., Eleid M. F., Michelena H. I. (2015). Effect of left ventricular ejection fraction on postoperative outcome in patients with severe aortic stenosis undergoing aortic valve replacement. *Circulation. Cardiovascular Imaging*.

[123] Taniguchi T., Morimoto T., Shiomi H. (2018). Prognostic impact of left ventricular ejection fraction in patients with severe aortic stenosis. *JACC. Cardiovascular Interventions*.

[124] Ito S., Miranda W. R., Nkomo V. T. (2018). Reduced left ventricular ejection fraction in patients with aortic stenosis. *Journal of the American College of Cardiology*.

[125] Bohbot Y., de Meester de Ravenstein C., Chadha G. (2019). Relationship between left ventricular ejection fraction and mortality in asymptomatic and minimally symptomatic patients with severe aortic stenosis. *JACC: Cardiovascular Imaging*.

[126] Pellikka P. A., Sarano M. E., Nishimura R. A. (2005). Outcome of 622 adults with asymptomatic, hemodynamically significant aortic stenosis during prolonged follow-up. *Circulation*.

[127] Lancellotti P., Donal E., Magne J. (2010). Risk stratification in asymptomatic moderate to severe aortic stenosis: the importance of the valvular, arterial and ventricular interplay. *Heart*.

[128] Kang D.-H., Park S.-J., Rim J. H. (2010). Early surgery versus conventional treatment in asymptomatic very severe aortic stenosis. *Circulation*.

[129] Tribouilloy C., Lévy F., Rusinaru D. (2009). Outcome after aortic valve replacement for low-flow/low-gradient aortic stenosis without contractile reserve on dobutamine stress echocardiography. *Journal of the American College of Cardiology*.

[130] Herrmann H. C., Pibarot P., Hueter I. (2013). Predictors of mortality and outcomes of therapy in low-flow severe aortic stenosis: a Placement of Aortic Transcatheter Valves (PARTNER) trial analysis. *Circulation*.

[131] Anjan V. Y., Herrmann H. C., Pibarot P. (2016). Evaluation of flow after transcatheter aortic valve replacement in patients with low-flow aortic stenosis: a secondary analysis of the PARTNER randomized clinical trial. *JAMA Cardiology*.

[132] Lopez-Marco A., Miller H., Youhana A. (2016). Low-flow low-gradient aortic stenosis: surgical outcomes and mid-term results after isolated aortic valve replacement. *European Journal of Cardio-Thoracic Surgery*.

[133] O’Sullivan C. J., Englberger L., Hosek N. (2015). Clinical outcomes and revascularization strategies in patients with low-flow, low-gradient severe aortic valve stenosis according to the assigned treatment modality. *JACC. Cardiovascular Interventions*.

[134] Nishimura R. A., Grantham J. A., Connolly H. M., Schaff H. V., Higano S. T., Holmes D. R. (2002). Low-output, low-gradient aortic stenosis in patients with depressed left ventricular systolic function: the clinical utility of the dobutamine challenge in the catheterization laboratory. *Circulation*.

[135] Monin J.-L., Quéré J. P., Monchi M. (2003). Low-gradient aortic stenosis: operative risk stratification and predictors for long-term outcome: a multicenter study using dobutamine stress hemodynamics. *Circulation*.

[136] Fougères E., Tribouilloy C., Monchi M. (2012). Outcomes of pseudo-severe aortic stenosis under conservative treatment. *European Heart Journal*.

[137] Eleid M. F., Padang R., al-Hijji M. (2019). Hemodynamic response in low-flow low-gradient aortic stenosis with preserved ejection fraction after TAVR. *Journal of the American College of Cardiology*.

[138] Rusinaru D., Bohbot Y., Ringle A., Maréchaux S., Diouf M., Tribouilloy C. (2018). Impact of low stroke volume on mortality in patients with severe aortic stenosis and preserved left ventricular ejection fraction. *European Heart Journal*.

[139] Zheng Q., Djohan A. H., Lim E. (2017). Effects of Aortic Valve Replacement on Severe Aortic Stenosis and Preserved Systolic Function: Systematic Review and Network Meta-analysis. *Scientific Reports*.

[140] Kang D.-H., Park S.-J., Lee S.-A. (2020). Early surgery or conservative care for asymptomatic aortic stenosis. *The New England Journal of Medicine*.

[141] Nakatsuma K., Taniguchi T., Morimoto T. (2019). B-type natriuretic peptide in patients with asymptomatic severe aortic stenosis. *Heart*.

[142] Lancellotti P., Magne J., Dulgheru R. (2018). Outcomes of patients with asymptomatic aortic stenosis followed up in heart valve clinics. *JAMA Cardiology*.

[143] Taniguchi T., Morimoto T., Shiomi H. (2018). Sudden death in patients with severe aortic stenosis: observations from the CURRENT AS registry. *Journal of the American Heart Association*.

[144] Rosenhek R., Zilberszac R., Schemper M. (2010). Natural history of very severe aortic stenosis. *Circulation*.

[145] Bergler-Klein J., Klaar U., Heger M. (2004). Natriuretic peptides predict symptom- free survival and postoperative outcome in severe aortic stenosis. *Circulation*.

[146] Gerber I. L., Stewart R. A. H., Legget M. E. (2003). Increased plasma natriuretic peptide levels reflect symptom onset in aortic stenosis. *Circulation*.

[147] LIM P., MONIN J., MONCHI M. (2004). Predictors of outcome in patients with severe aortic stenosis and normal left ventricular function: role of B-type natriuretic peptide. *European Heart Journal*.

[148] Taniguchi T., Morimoto T., Shiomi H. (2015). Initial surgical versus conservative strategies in patients with asymptomatic severe aortic stenosis. *Journal of the American College of Cardiology*.

[149] Nishimura S., Izumi C., Nishiga M. (2016). Predictors of rapid progression and clinical outcome of asymptomatic severe aortic stenosis. *Circulation Journal*.

[150] Walther T., Blumenstein J., van Linden A., Kempfert J. (2012). Contemporary management of aortic stenosis: surgical aortic valve replacement remains the gold standard. *Heart*.

